# Joint associations of sleep quality and depressive symptoms with incident digestive disease in a nationally representative Chinese cohort

**DOI:** 10.1038/s41598-026-56067-7

**Published:** 2026-06-05

**Authors:** Wenjie Ke, Cong Peng, Qi Lu

**Affiliations:** 1 Department of Hernia and Abdominal Wall Surgery, Tongji Medical College, The Central Hospital of Wuhan, Huazhong University of Science and Technology, Wuhan, China; 2 Department of Otolaryngology, Guizhou Provincial People’s Hospital, Guiyang, 550002 Guizhou China; 3 School of Clinical Medicine, Guizhou Medical University, Guiyang, 550004 Guizhou China; 4 Department of Hepatobiliary Surgery, Huangshi Central Hospital (Affiliated Hospital of Hubei Polytechnic University), Huangshi, China

**Keywords:** Sleep quality, Depressive symptoms, Incident digestive disease, China Health and Retirement Longitudinal Study, Prospective cohort study, Diseases, Gastroenterology, Health care, Medical research, Psychology, Psychology, Risk factors

## Abstract

Sleep disturbance and depressive symptoms are common and modifiable, yet their joint and longitudinal associations with incident digestive disease remain unclear. Using data from the China Health and Retirement Longitudinal Study, this national prospective cohort study included 3,711 Chinese adults free of digestive disease at baseline. Sleep quality (SQ) was derived from questionnaire-based sleep items, and depressive symptoms were assessed using the 10-item Center for Epidemiologic Studies Depression Scale (CESD-10). Cox proportional hazards models estimated associations with incident self-reported digestive disease after adjustment for sociodemographic, lifestyle, and comorbidity factors. Exploratory mediation analyses assessed model-based indirect associations, and joint trajectory-pattern analyses characterized longitudinal patterns of SQ and depressive symptoms. Sensitivity analyses examined alternative exposure definitions, restriction to participants without baseline comorbidities, and modified CESD-9 analyses after excluding the restless-sleep item. In separate models, poor SQ (HR = 1.67, 95% CI 1.39–2.01) and depressive symptoms (HR = 1.33, 95% CI 1.13–1.57) were both associated with higher risk. Compared with good SQ without depressive symptoms, elevated risks were observed for poor SQ without depressive symptoms (fully adjusted HR = 1.91, 95% CI 1.51–2.42) and poor SQ with depressive symptoms (HR = 1.80, 95% CI 1.45–2.23). Exploratory mediation analyses showed an attenuated indirect association through depressive symptoms for the SQ–digestive disease association after full adjustment (ACME = 0.006; *P* = 0.106; proportion mediated = 7.8%), whereas the indirect association through SQ for the depressive symptoms–digestive disease association remained evident (ACME = 0.025; proportion mediated = 47.3%). Joint trajectory-pattern analyses showed elevated risks in the moderate worsening and marked worsening/lower SQ groups (HR = 1.50, 95% CI 1.24–1.82; and HR = 1.48, 95% CI 1.23–1.78, respectively). Sensitivity analyses were generally consistent, including analyses using modified CESD-9 after excluding the restless-sleep item. Poor SQ and depressive symptoms were associated with higher subsequent risk of incident self-reported digestive disease. The findings highlight a potential sleep–mood–digestive health link, although causal and interventional implications require further validation using objective sleep measures and refined disease phenotyping.

**Supplementary Information:**

The online version contains supplementary material available at 10.1038/s41598-026-56067-7.

## Introduction

Digestive system diseases impose a substantial and growing burden on global health. In 2019, they accounted for an estimated 88.99 million disability-adjusted life years (DALYs), representing 3.51% of all DALYs and ranking as the 13th leading cause of health loss worldwide^1^. From 1990 to 2019, incident cases, deaths, and DALYs attributable to digestive diseases rose by ~ 74%, 38%, and 23%, respectively^2^. The epidemiology is also shifting: inflammatory bowel disease (IBD), once considered predominantly a Western condition, has surged in newly industrialized regions of Asia, Africa, and Latin America^3^. The etiology of digestive diseases is multifactorial, involving genetic susceptibility, diet, infections, gut microbiota dysbiosis, and psychosocial influences^4–7^.

Within this context, behavioral and mental-health factors have gained attention as modifiable determinants of gastrointestinal disorders^8–10^. Poor sleep quality (SQ) and depressive symptoms, both common and potentially modifiable, have been associated with adverse gastrointestinal outcomes. In the UK Biobank study of approximately 410,000 adults, the highest composite healthy-sleep score was associated with a 28% lower risk of incident digestive disease, whereas poor sleep combined with high genetic susceptibility was associated with an approximately 60% higher risk^11^. In an Asian population-based cohort including 45,310 individuals with sleep disorders and 90,620 controls, sleep disorder was strongly associated with incident functional dyspepsia during a mean follow-up of approximately 10 years (adjusted hazard ratio [HR] 3.30, 95% confidence interval [CI] 2.82–3.89)^12^. Evidence regarding mood-related factors is also accumulating: a meta-analysis of ~ 9 million participants across 13 cohorts reported elevated IBD risk among individuals with prior depression, with relative risks of 1.17 (95% CI 1.02–1.34) for Crohn’s disease and 1.21 (95% CI 1.10–1.33) for ulcerative colitis^13^, and Mendelian randomization analyses have linked depression liability to several gastrointestinal diseases, including non-alcoholic fatty liver disease, gastroesophageal reflux disease, chronic pancreatitis, and ulcerative colitis^14^. SQ disturbances and depressive symptoms frequently co-occur. Up to 75% of patients with depression report insomnia symptoms, insomnia has been associated with an approximately two-fold higher future risk of depression (odds ratio 2.10, 95% CI 1.59–2.77), and short or poor-quality sleep predicts new-onset depressive symptoms in large Chinese cohorts^15–20^. Cohort data further suggest that concurrent insomnia and depression may be associated with a 2–3-fold higher functional dyspepsia risk than insomnia alone^12^. However, longitudinal evidence on the joint-category and trajectory-pattern associations of SQ and depressive symptoms with digestive-health outcomes remains limited.

To address this gap, data from the China Health and Retirement Longitudinal Study (CHARLS) were used to evaluate the prospective associations of SQ, depressive symptoms, and their joint categories with incident digestive disease. Exploratory mediation analyses and joint trajectory-pattern analyses were further applied to characterize potential model-based indirect associations and longitudinal co-occurrence patterns. These analyses aimed to provide population-based evidence for the sleep–mood–digestive health relationship and to inform observational risk assessment in middle-aged and older adults.

## Methods and materials

### Study design and data source

This study used data from the CHARLS, a nationally representative cohort of community-dwelling adults aged ≥ 45 years and their spouses in mainland China^21^. CHARLS employs face-to-face, computer-assisted personal interviewing to collect harmonised information on sociodemographics, health behaviours, clinical conditions, and physical/laboratory measurements. For the present prospective analysis, the 2011–2012 baseline and the 2013, 2015, and 2018 follow-up waves were assembled. The CHARLS protocol was approved by the Peking University Institutional Review Board (ethics approval IRB00001052-11015). All participants provided written informed consent. The analysis used de-identified data and complied with the Declaration of Helsinki.

### Study population and eligibility criteria

**Fig. 1:**
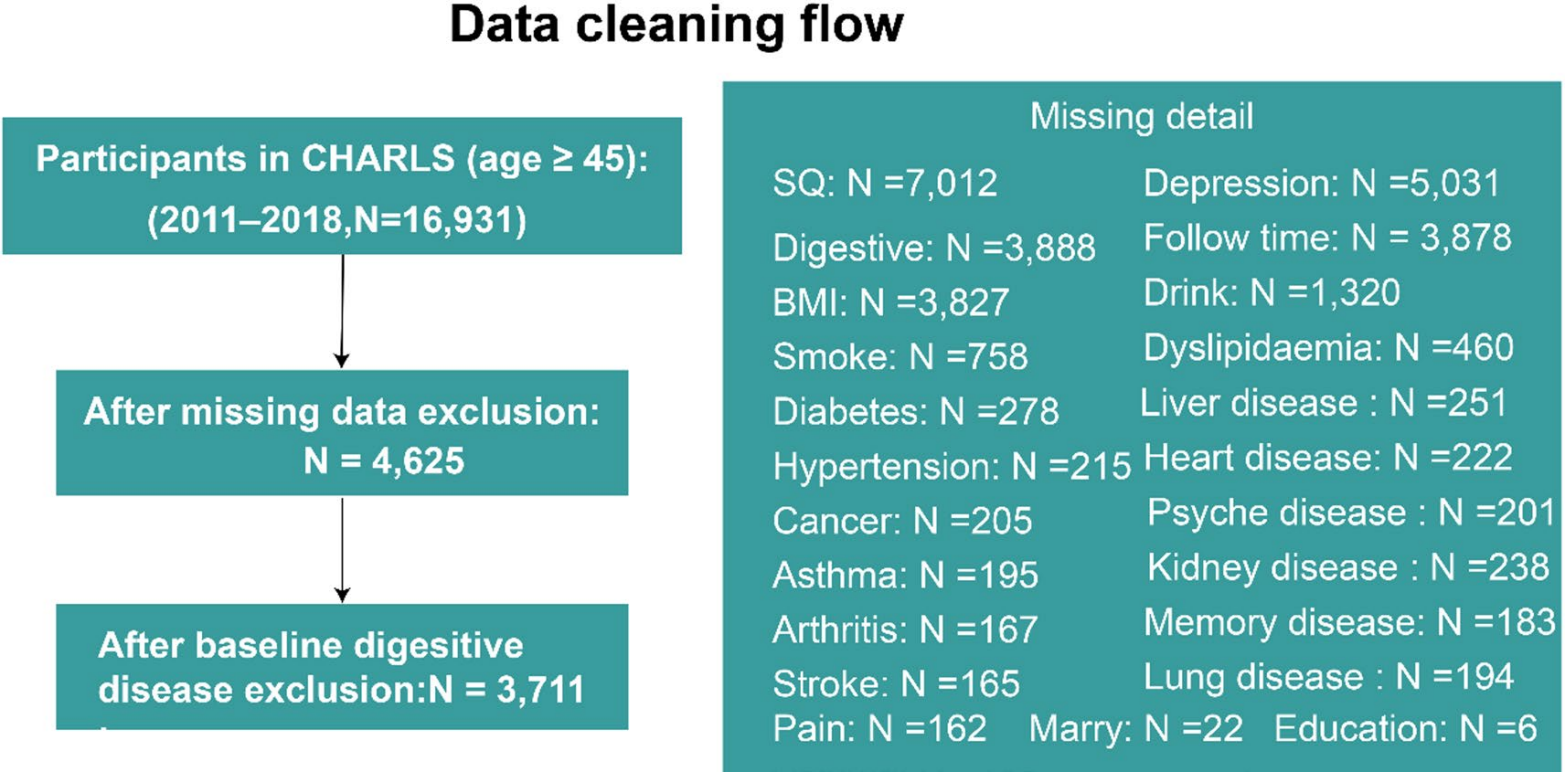
Data cleaning and participant selection flowchart. Participants aged ≥ 45 years from CHARLS 2011–2018 were first screened. Participants with missing information on key exposures, covariates, or follow-up/outcome ascertainment were excluded, leaving 4,625 participants. Because missingness overlapped across variables, variable-specific missing counts are shown as descriptive details and are not additive. Participants with self-reported physician-diagnosed digestive disease at baseline were then excluded, yielding a final analytic cohort of 3,711 participants. CHARLS, China Health and Retirement Longitudinal Study; BMI, body mass index; SQ, sleep quality; CESD-10, 10-item Center for Epidemiologic Studies Depression Scale.

Participants were eligible if, at baseline (2011–2012), they completed the core health questionnaire and had complete data on self-rated SQ and depressive symptoms. Incident outcomes were ascertained by linking records from the 2013, 2015, and 2018 follow-up waves. Exclusions were: (i) self-reported, physician-diagnosed stomach or other digestive diseases at baseline; (ii) missing key covariates (e.g., age, sex, marital status, education, residence, smoking, drinking, body mass index) not amenable to analysis; and (iii) insufficient follow-up information to determine outcome status. The final analytic cohort comprised 3,711 participants. The index date was the baseline interview; event time was calculated from baseline to the first report of incident digestive disease. Participants without an event were censored at the date of last contact or loss to follow-up. A schematic of data processing and exclusions is provided in Fig. 1.

### Self-rated sleep quality (SQ)

SQ was operationalised as a two-component composite. First, restless sleep was taken from the 10-item Center for Epidemiologic Studies Depression Scale (CESD-10) item “*My sleep was restless*” with response options “<1 day,” “1–2 days,” “3–4 days,” and “5–7 days.” Responses were initially scored 1–4 (higher = more frequent restlessness) and then reverse-coded to 0–3 so that 3 = < 1 day, 2 = 1–2 days, 1 = 3–4 days, and 0 = 5–7 days (higher values denote better sleep calmness). Second, mean night-time sleep duration over the past month was categorised as ≤ 6 h, 7–8 h, or > 8 h and recoded to a binary indicator (1 for normal 7–8 h; 0 for abnormal ≤ 6 h or > 8 h). The reverse-coded restlessness score (0–3) and the duration indicator (0–1) were summed to yield a composite SQ index ranging from 0 to 4, with higher scores indicating better overall SQ. For categorical analyses, SQ was classified as poor (< 3), moderate (= 3), and good (= 4)^22^.

### Depressive symptoms

Depressive symptoms were assessed with the CESD-10, referencing the past 7 days. Each item uses a four-level frequency response including “<1 day,” “1–2 days,” “3–4 days,” “5–7 days”—scored 0–3 so that higher values reflect more frequent symptoms. As per instrument specifications, positive affect items are reverse-coded. Item scores are summed to yield a total score of 0–30, with higher totals indicating greater depressive symptom burden. For analysis, CESD-10 was used in two ways: (i) as a binary indicator of depressive symptoms, defined as CESD-10 ≥ 10, a threshold widely applied and validated in Chinese middle-aged and older adults^23^; and (ii) as a continuous score, modelled both per 1-SD increase (SD = 5.66) and with restricted cubic splines (RCS) to characterise dose–response and test for non-linearity. In joint-category models, the binary CESD-10 indicator was cross-classified with SQ. In exploratory mediation analyses, CESD-10 served as either the mediator of the SQ-digestive disease association or the exposure in the alternative direction, according to the direction being evaluated. Because the SQ measure incorporated the restless-sleep item from the CESD-10 scale, additional sensitivity analyses used a modified CESD-9 score calculated by subtracting the restless-sleep item from the CESD-10 total score. The modified CESD-9 score was used only for sensitivity analyses and was not interpreted as a clinical diagnosis of depression. To ensure reproducibility, a complete-case approach was applied for CESD-10: participants with any missing CESD-10 items were excluded from analyses that used the scale.

### Incident digestive disease

Incident digestive disease was defined as the first follow-up report of a physician-diagnosed “*stomach or other digestive disease*,” excluding benign tumours and cancers. Ascertainment relied on self- or proxy-report to the standard CHARLS question, “*Has a doctor ever told you that you have …* ?” This case definition follows the CHARLS protocol for chronic conditions and aligns with prior applications of the cohort.

### Covariates

Baseline covariates were collected at the 2011–2012 interview using interviewer-administered questionnaires and standardised measurements. Sociodemographic variables included age (years; modelled continuously), sex (male/female), educational attainment (illiterate, literate, elementary school, middle school, high school or above), marital status (married vs. other), and residential setting (urban community vs. rural village). Lifestyle factors comprised smoking status (never, former, current) and alcohol consumption (never, former, current), both self-reported at baseline. Adiposity was assessed by body mass index (BMI), calculated as weight (kg)/height (m)^2 from standardised measurements and categorised according to Chinese adult cut points: **<**18.5 kg/m² (underweight), 18.5–23.9 (normal), 24.0–27.9 (overweight), and ≥ 28.0 (obesity). Physician-diagnosed comorbidities were ascertained by self- or proxy-report to the standard question “*Has a doctor ever told you that you have … ?*”, including hypertension, diabetes, dyslipidaemia, coronary/heart disease, liver disease, lung disease, stroke, kidney disease, asthma, arthritis, pain, memory disorder, and psychiatric disorder; each was coded yes/no. Reference categories for categorical covariates are specified in the corresponding tables. All covariates were defined at baseline prior to outcome ascertainment.

### Statistical analysis

All analyses followed a prespecified plan with two-sided tests and α = 0.05. A complete-case approach was used, restricting each model to participants with non-missing values for all variables included. Continuous variables were summarised as mean (SD) or median (IQR), and categorical variables as n (%). Between-group differences across sleep-quality (SQ) or depressive-symptom strata were assessed using ANOVA (or Kruskal–Wallis for non-normal distributions) and **χ²** tests for categorical variables. Analyses were conducted in R (version 4.3.2; R Foundation for Statistical Computing, Vienna, Austria). Key packages and versions were: survival 3.5-7 (Cox proportional hazards models and diagnostics); rms 6.7-1 (RCS within Cox models); mediation 4.5.0 (mediation analyses); lcmm 1.9.7 (multivariate/parallel-process latent-class trajectory modelling via *multlcmm*); ggplot2 3.4.4 and dplyr 1.1.4 (data management and visualisation).

### Time-to-event modelling

Associations with incident digestive disease were estimated using Cox proportional hazards models with months as the time scale. Entry time was the baseline interview date; censoring occurred at last contact, loss to follow-up, or end of observation. Hazard ratios (HRs) with 95% confidence intervals (CIs) were reported. Four hierarchical models were fitted: Model 0, unadjusted; Model 1, adjusted for age and sex; Model 2, additionally adjusted for education, residence, marital status, BMI category, smoking status, and drinking status; and Model 3, additionally adjusted for hypertension, diabetes, dyslipidaemia, heart disease, liver disease, lung disease, stroke, kidney disease, asthma, arthritis, pain, memory disorder, and psychiatric disorder.

### Dose–response and non-linearity

SQ score and CESD-10 score were modelled separately in relation to incident digestive disease using RCS within Cox models. HRs and 95% CIs were estimated across the observed range under the same four adjustment sets. Wald tests were used to evaluate the overall association and the joint contribution of non-linear spline terms.

### Joint-category analyses of SQ and depressive symptoms

SQ category (good/moderate/poor) was cross-classified with depressive symptom status (CESD-10 ≥ 10 vs. < 10), using good SQ without depressive symptoms as the reference group. HRs and 95% CIs were estimated for each joint category using the same adjustment sets. These analyses were interpreted as joint-category associations and were not considered formal tests of additive or multiplicative interaction.

### Exploratory mediation analyses

Exploratory mediation analyses were performed to examine potential indirect associations between SQ, depressive symptoms, and incident digestive disease. Using standard mediation terminology, the average causal mediation effect (ACME), average direct effect (ADE), total effect (TE), and proportion mediated (PM) were estimated. Two directions were evaluated: depressive symptoms as the mediator of the SQ–digestive disease association, and SQ as the mediator of the depressive symptoms–digestive disease association. Mediator and outcome regression models were fitted according to the scale of the mediator and outcome, and uncertainty was quantified using simulation or nonparametric bootstrapping. All four adjustment sets were applied. These analyses were exploratory and assumption-dependent and were not interpreted as confirmation of causal pathways.

### Joint trajectory modelling

To characterize parallel longitudinal patterns of SQ and depressive symptoms, joint trajectory-pattern analysis was applied to repeated SQ and CESD-10 measures from the 2011 baseline and the 2015 and 2018 follow-up waves. A three-group solution was retained based on model fit, interpretability, and classification adequacy. Trajectory membership was then entered as the exposure in Cox proportional hazards models, with the group characterized by stable/low depressive symptoms with higher SQ serving as the reference group. The same covariate adjustment sets were applied.

### Sensitivity analyses

Several sensitivity analyses were conducted to assess robustness. Alternative exposure definitions included modelling SQ as a continuous exposure, modelling CESD-10 per 1-SD increase, recoding SQ as moderate/poor versus good, and applying an alternative depressive symptom threshold of CESD-10 ≥ 12. Analyses were also repeated among participants without baseline comorbidities. To address potential item overlap between SQ and CESD-10, a modified CESD-9 score was calculated by subtracting the restless-sleep item from the CESD-10 total score. Using modified CESD-9, baseline association analyses, Cox models, joint-category analyses, dose–response analyses, exploratory bidirectional mediation analyses, and joint trajectory analyses were repeated. High modified CESD-9 burden was defined as modified CESD-9 score ≥ 9 for sensitivity analyses only and was not interpreted as a clinical diagnosis of depression. The proportional hazards assumption was assessed using Schoenfeld residuals for the main Cox models and sensitivity models.

## Results

### Baseline characteristics

Among 3,711 participants (Good 2,227; Moderate 611; Poor 873), mean age was similar across groups (57.5–58.2 years, *P* = 0.099). The proportion of females increased and marriage slightly declined with poorer SQ (*P* ≤ 0.032). Education showed a clear gradient, with higher illiteracy and lower high-school attainment (*P* < 0.001), while urban residence was comparable. Smoking and drinking were less common, whereas underweight and obesity were more prevalent with poorer SQ (all *P* ≤ 0.002). Depressive symptom burden rose markedly across SQ categories (median CESD-10 from 4 to 11; prevalence of depressive symptoms 14.1% to 41.6%; *P* < 0.001). Comorbidities including heart and lung diseases, arthritis, and pain were more frequent with poorer SQ (*P* ≤ 0.006). Incident digestive disease increased progressively (15.5% to 28.1%; *P* < 0.001; Table 1). By depressive symptom status, participants with depressive symptoms (*n* = 1,018) had less favorable sociodemographic, lifestyle, and comorbidity profiles than those without depressive symptoms (*n* = 2,693), and had a higher incidence of digestive disease (26.2% vs. 16.9%; *P* < 0.001; Table 2).

**Table 1:** Baseline characteristics by SQ at baseline.

Characteristic	Overall (*n* = 3711)	Good (*n* = 2227)	Moderate (*n* = 611)	Poor (*n* = 873)	*P* value
**Age**,** years**	57.71 (8.23)	57.53 (8.29)	57.64 (7.84)	58.23 (8.32)	0.099
**Sex**,** n (%)**					< 0.001
Female	1824 (49.2)	988 (44.4)	336 (55.0)	500 (57.3)	
Male	1887 (50.8)	1239 (55.6)	275 (45.0)	373 (42.7)	
**Marital status**,** n (%)**					0.032
Married	3388 (91.3)	2055 (92.3)	551 (90.2)	782 (89.6)	
Other	323 (8.7)	172 (7.7)	60 (9.8)	91 (10.4)	
**Education level**,** n (%)**					< 0.001
Illiterate	888 (23.9)	477 (21.4)	158 (25.9)	253 (29.0)	
Literate	683 (18.4)	406 (18.2)	105 (17.2)	172 (19.7)	
Elementary school	876 (23.6)	533 (23.9)	134 (21.9)	209 (23.9)	
Middle school	828 (22.3)	511 (22.9)	142 (23.2)	175 (20.0)	
High school or above	436 (11.7)	300 (13.5)	72 (11.8)	64 (7.3)	
**Residence**,** n (%)**					0.158
Urban community	1266 (34.1)	744 (33.4)	229 (37.5)	293 (33.6)	
Rural village	2445 (65.9)	1483 (66.6)	382 (62.5)	580 (66.4)	
**Smoking status**,** n (%)**					< 0.001
Current	1207 (32.5)	775 (34.8)	164 (26.8)	268 (30.7)	
Ever	311 (8.4)	202 (9.1)	49 (8.0)	60 (6.9)	
Never	2193 (59.1)	1250 (56.1)	398 (65.1)	545 (62.4)	
**Drinking status**,** n (%)**					0.002
Current	1216 (32.8)	784 (35.2)	189 (30.9)	243 (27.8)	
Ever	293 (7.9)	176 (7.9)	46 (7.5)	71 (8.1)	
Never	2202 (59.3)	1267 (56.9)	376 (61.5)	559 (64.0)	
**BMI category**,** n (%)**					< 0.001
Underweight (< 18.5)	182 (4.9)	87 (3.9)	31 (5.1)	64 (7.3)	
Normal (18.5–23.9)	1940 (52.3)	1164 (52.3)	309 (50.6)	467 (53.5)	
Overweight (24.0–27.9)	1118 (30.1)	715 (32.1)	189 (30.9)	214 (24.5)	
Obesity (≥ 28.0)	471 (12.7)	261 (11.7)	82 (13.4)	128 (14.7)	
**CESD-10 score**,** median [IQR]**	6.00 [3.00, 10.00]	4.00 [1.00, 7.00]	7.00 [4.00, 11.00]	11.00 [7.00, 15.00]	< 0.001
**Depressive symptoms (CESD-10 ≥ 10)**,** n (%)**					< 0.001
No	2693 (72.6)	1912 (85.9)	418 (68.4)	363 (41.6)	
Yes	1018 (27.4)	315 (14.1)	193 (31.6)	510 (58.4)	
**Hypertension**,** n (%)**					< 0.001
No	2842 (76.6)	1761 (79.1)	433 (70.9)	648 (74.2)	
Yes	869 (23.4)	466 (20.9)	178 (29.1)	225 (25.8)	
**Diabetes**,** n (%)**					0.034
No	3507 (94.5)	2119 (95.2)	565 (92.5)	823 (94.3)	
Yes	204 (5.5)	108 (4.8)	46 (7.5)	50 (5.7)	
**Dyslipidaemia**,** n (%)**					< 0.001
No	3421 (92.2)	2069 (92.9)	536 (87.7)	816 (93.5)	
Yes	290 (7.8)	158 (7.1)	75 (12.3)	57 (6.5)	
**Heart disease**,** n (%)**					0.006
No	3397 (91.5)	2063 (92.6)	556 (91.0)	778 (89.1)	
Yes	314 (8.5)	164 (7.4)	55 (9.0)	95 (10.9)	
**Liver disease**,** n (%)**					0.323
No	3623 (97.6)	2181 (97.9)	594 (97.2)	848 (97.1)	
Yes	88 (2.4)	46 (2.1)	17 (2.8)	25 (2.9)	
**Lung disease**,** n (%)**					0.001
No	3420 (92.2)	2079 (93.4)	561 (91.8)	780 (89.3)	
Yes	291 (7.8)	148 (6.6)	50 (8.2)	93 (10.7)	
**Stroke**,** n (%)**					0.012
No	3644 (98.2)	2198 (98.7)	593 (97.1)	853 (97.7)	
Yes	67 (1.8)	29 (1.3)	18 (2.9)	20 (2.3)	
**Chronic kidney disease**,** n (%)**					0.147
No	3562 (96.0)	2149 (96.5)	581 (95.1)	832 (95.3)	
Yes	149 (4.0)	78 (3.5)	30 (4.9)	41 (4.7)	
**Asthma**,** n (%)**					0.289
No	3567 (96.1)	2149 (96.5)	586 (95.9)	832 (95.3)	
Yes	144 (3.9)	78 (3.5)	25 (4.1)	41 (4.7)	
**Arthritis**,** n (%)**					< 0.001
No	2742 (73.9)	1726 (77.5)	439 (71.8)	577 (66.1)	
Yes	969 (26.1)	501 (22.5)	172 (28.2)	296 (33.9)	
**Pain**,** n (%)**					< 0.001
No	2767 (74.6)	1800 (80.8)	439 (71.8)	528 (60.5)	
Yes	944 (25.4)	427 (19.2)	172 (28.2)	345 (39.5)	
**Memory-related disease**,** n (%)**					0.766
No	3683 (99.2)	2212 (99.3)	606 (99.2)	865 (99.1)	
Yes	28 (0.8)	15 (0.7)	5 (0.8)	8 (0.9)	
**Psychiatric disease**,** n (%)**					0.098
No	3689 (99.4)	2218 (99.6)	604 (98.9)	867 (99.3)	
Yes	22 (0.6)	9 (0.4)	7 (1.1)	6 (0.7)	
**Incident digestive disease during follow-up**,** n (%)**					< 0.001
No	2989 (80.5)	1882 (84.5)	479 (78.4)	628 (71.9)	
Yes	722 (19.5)	345 (15.5)	132 (21.6)	245 (28.1)	

BMI, body mass index; CESD-10, 10-item Center for Epidemiologic Studies Depression Scale; SQ, sleep quality; IQR, interquartile range.

**Table 2:** Baseline characteristics by depressive symptom status at baseline.

Characteristic	Overall (*n* = 3,711)	No (*n* = 2,693)	Yes (*n* = 1,018)	*P* value
**Age**,** years**	57.71 (8.23)	57.42 (8.30)	58.48 (8.01)	< 0.001
**Sex**,** n (%)**				< 0.001
Female	1824 (49.2)	1234(45.8)	590 (58.0)	
Male	1887 (50.8)	1459 (54.2)	428 (42.0)	
**Marital status**,** n (%)**				< 0.001
Married	3388 (91.3)	2488 (92.4)	900 (88.4)	
Other	323 (8.7)	205 (7.6)	118 (11.6)	
**Education level**,** n (%)**				< 0.001
Illiterate	888 (23.9)	577 (21.4)	311 (30.6)	
Literate	683 (18.4)	453 (16.8)	230 (22.6)	
Elementary school	876 (23.6)	640 (23.8)	236 (23.2)	
Middle school	828 (22.3)	648 (24.1)	180 (17.7)	
High school or above	436 (11.7)	375 (13.9)	61 (6.0)	
**Residence**,** n (%)**				< 0.001
Urban community	1266 (34.1)	992 (36.8)	274 (26.9)	
Rural village	2445 (65.9)	1701 (63.2)	744 (73.1)	
**Smoking status**,** n (%)**				0.098
Current	1207 (32.5)	901 (33.5)	306 (30.1)	
Ever	311 (8.4)	229 (8.5)	82 (8.1)	
Never	2193 (59.1)	1563 (58.0)	630 (61.9)	
**Drinking status**,** n (%)**				< 0.001
Current	1216 (32.8)	953 (35.4)	263 (25.8)	
Ever	293 (7.9)	179 (6.6)	114 (11.2)	
Never	2202 (59.3)	1561 (58.0)	641 (63.0)	
**BMI category**,** n (%)**				< 0.001
Underweight (< 18.5)	182 (4.9)	102 (3.8)	80 (7.9)	
Normal (18.5–23.9)	1940 (52.3)	1385 (51.4)	555 (54.5)	
Overweight (24.0–27.9)	1118 (30.1)	859 (31.9)	259 (25.4)	
Obesity (≥ 28.0)	471 (12.7)	347 (12.9)	124 (12.2)	
**CESD-10 score**,** median [IQR]**	6.00 [3.00, 10.00]	4.00 [2.00, 6.00]	13.00 [11.00, 16.75]	< 0.001
**Depressive symptoms (CESD-10 ≥ 10)**,** n (%)**				—
No	2693 (72.6)	2693 (100.0)	0 (0.0)	
Yes	1018 (27.4)	0 (0.0)	1018 (100.0)	
**Hypertension**,** n (%)**				0.011
No	2842 (76.6)	2092 (77.7)	750 (73.7)	
Yes	869 (23.4)	601 (22.3)	268 (26.3)	
**Diabetes**,** n (%)**				0.124
No	3507 (94.5)	2555 (94.9)	952 (93.5)	
Yes	204 (5.5)	138 (5.1)	66 (6.5)	
**Dyslipidaemia**,** n (%)**				0.498
No	3421 (92.2)	2488 (92.4)	933 (91.7)	
Yes	290 (7.8)	205 (7.6)	85 (8.3)	
**Heart disease**,** n (%)**				0.001
No	3397 (91.5)	2490 (92.5)	907 (89.1)	
Yes	314 (8.5)	203 (7.5)	111 (10.9)	
**Liver disease**,** n (%)**				0.931
No	3623 (97.6)	2630 (97.7)	993 (97.5)	
Yes	88 (2.4)	63 (2.3)	25 (2.5)	
**Lung disease**,** n (%)**				< 0.001
No	3420 (92.2)	2519 (93.5)	901 (88.5)	
Yes	291 (7.8)	174 (6.5)	117 (11.5)	
**Stroke**,** n (%)**				0.001
No	3644 (98.2)	2657 (98.7)	987 (97.0)	
Yes	67 (1.8)	36 (1.3)	31 (3.0)	
**Chronic kidney disease**,** n (%)**				0.106
No	3562 (96.0)	2594 (96.3)	968 (95.1)	
Yes	149 (4.0)	99 (3.7)	50 (4.9)	
**Asthma**,** n (%)**				< 0.001
No	3567 (96.1)	2609 (96.9)	958 (94.1)	
Yes	144 (3.9)	84 (3.1)	60 (5.9)	
**Arthritis**,** n (%)**				< 0.001
No	2742 (73.9)	2104 (78.1)	638 (62.7)	
Yes	969 (26.1)	589 (21.9)	380 (37.3)	
**Pain**,** n (%)**				< 0.001
No	2767 (74.6)	2225 (82.6)	542 (53.2)	
Yes	944 (25.4)	468 (17.4)	476 (46.8)	
**Memory-related disease**,** n (%)**				0.439
No	3683 (99.2)	2675 (99.3)	1008 (99.0)	
Yes	28 (0.8)	18 (0.7)	10 (1.0)	
**Psychiatric disease**,** n (%)**				< 0.001
No	3689 (99.4)	2686 (99.7)	1003 (98.5)	
Yes	22 (0.6)	(0.3)	15 (1.5)	
**Incident digestive disease during follow-up**,** n (%)**				< 0.001
No	2989 (80.5)	2238 (83.1)	751 (73.8)	
Yes	722 (19.5)	455 (16.9)	267 (26.2)	

BMI, body mass index; CESD-10, 10-item Center for Epidemiologic Studies Depression Scale; IQR, interquartile range.

### Association between SQ and depressive symptoms

**Fig. 2:**
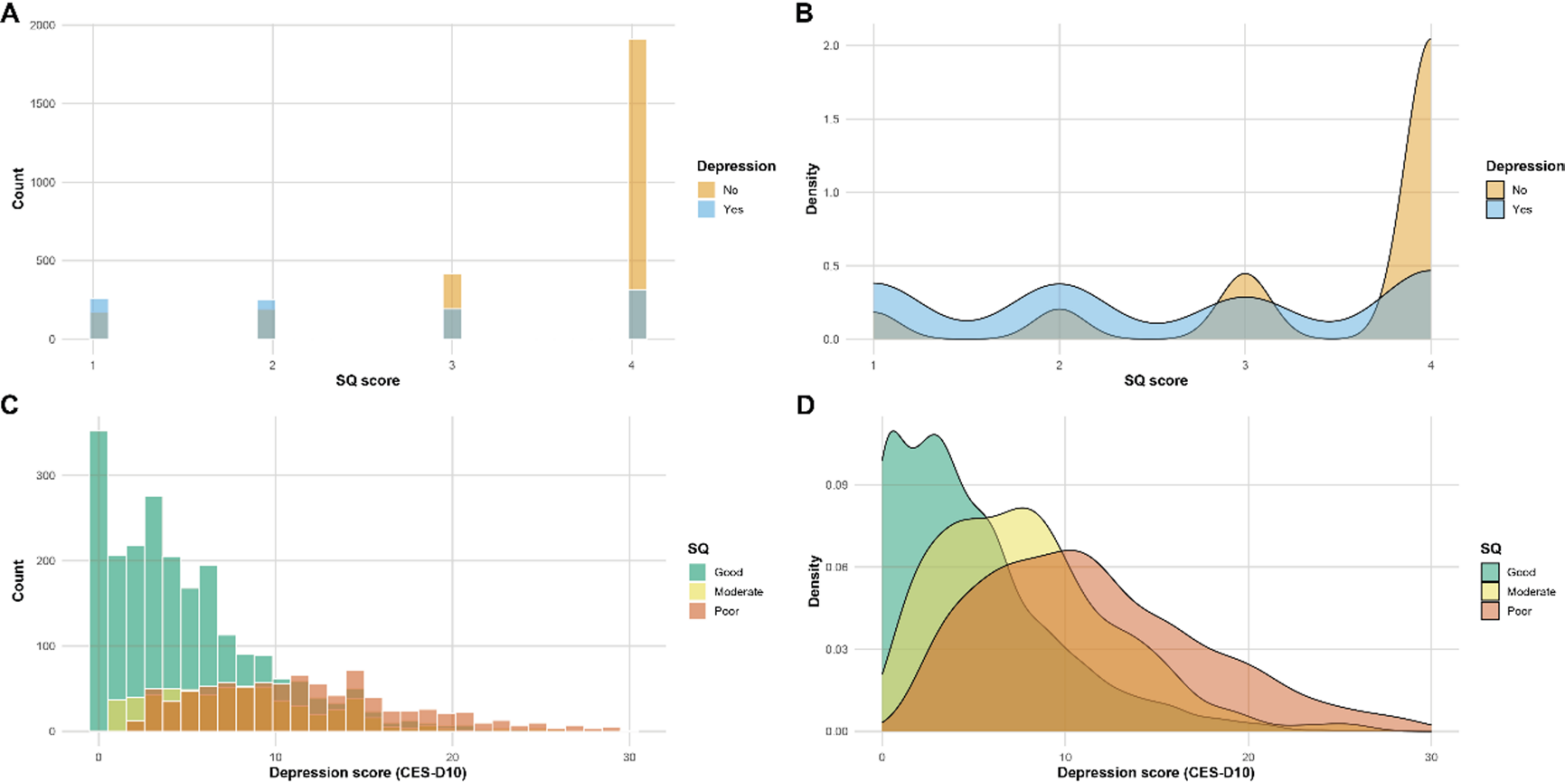
Distribution of SQ and depressive symptom levels in the study population. (A–B) Distribution and density of SQ scores stratified by depressive symptom status. Participants with depressive symptoms (CESD-10 ≥ 10) exhibited a right-shifted distribution toward poorer SQ. (C–D) Distribution and density of depressive symptom scores (CESD-10) stratified by SQ categories. SQ, sleep quality; CESD-10, 10-item Center for Epidemiologic Studies Depression Scale.

The baseline relationship between SQ and depressive symptoms was examined. Participants with depressive symptoms had lower SQ scores than those without depressive symptoms (mean 2.56 vs. 3.51; median 2 vs. 4; Supplementary Table 1). Conversely, depressive symptom burden increased across SQ categories, with mean CESD-10 scores of 4.85, 7.90, and 11.60 and depressive symptom prevalence of 14.1%, 31.6%, and 41.6% in the good, moderate, and poor SQ groups, respectively (Fig. 2, Supplementary Table 2). Distribution plots showed a consistent inverse relationship between SQ and depressive symptoms. RCS models showed a significant nonlinear association between CESD-10 and SQ (*P*-overall < 0.001; *P*-nonlinear ≈ 0.003–0.004; Supplementary Fig. 1; Supplementary Table 3). This association remained evident after sequential adjustment for demographics, sociodemographics, and lifestyle/comorbidities, with only modest attenuation.

### Associations of SQ and depressive symptoms with incident digestive disease

**Fig. 3:**
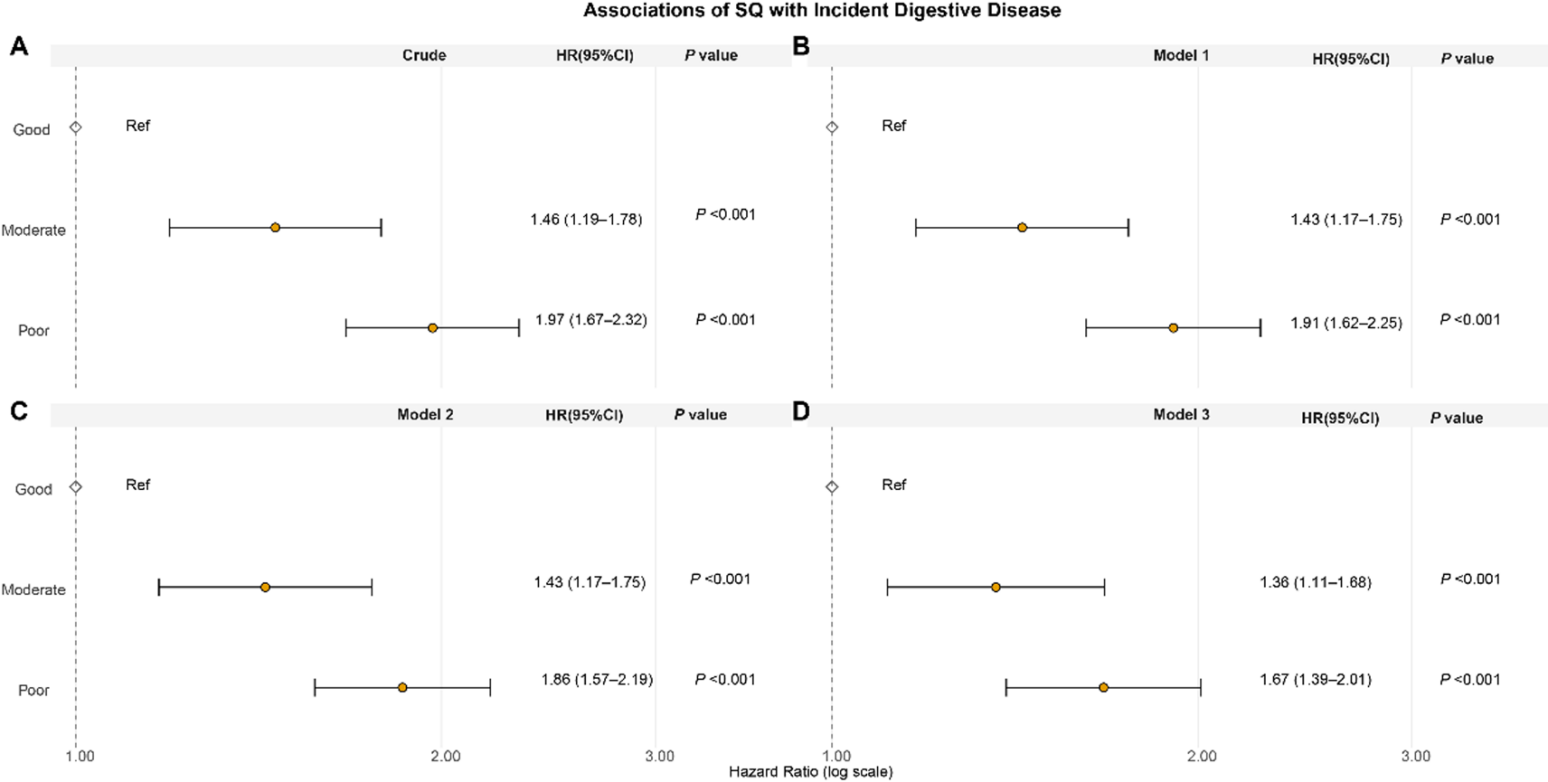
Associations of SQ with incident digestive disease. Forest plots displaying the HRs and 95% CIs for the association between SQ and incident digestive disease across four models: (**A**) Crude model; (**B**) Model 1 adjusted for age and sex; (**C**) Model 2 further adjusted for sociodemographic factors (education level, residence, and marital status, BMI, smoking, and drinking status); and (**D**) Model 3 additionally adjusted for comorbidities including hypertension, diabetes, dyslipidemia, heart disease, liver disease, lung disease, stroke, kidney disease, asthma, arthritis, pain, memory disorder, and psychological disorder. Participants with good SQ served as the reference group. HRs > 1 indicate increased risk of incident digestive disease. HRs, hazard ratios; CIs, confidence intervals; SQ, sleep quality.

**Fig. 4:**
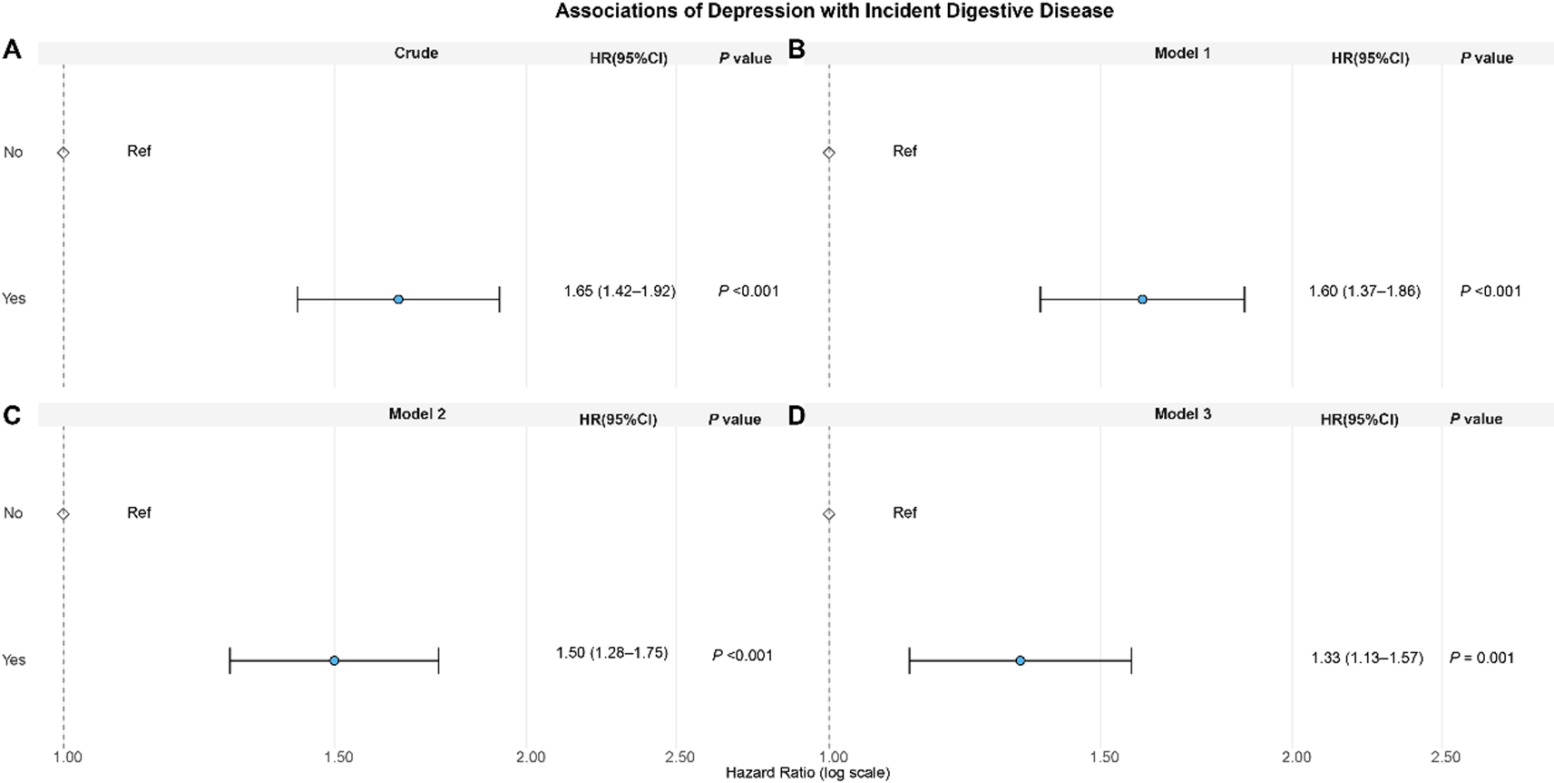
Associations of depressive symptoms with incident digestive disease. Forest plots showing HRs (95% CIs) for incident digestive disease according to depressive symptoms (CESD-10 ≥ 10 = Yes). (**A**) Crude model; (**B**) Model 1 adjusted for age and sex; (**C**) Model 2 further adjusted for sociodemographic variables (education level, residence, marital status, BMI, smoking, and drinking); and (**D**) Model 3 additionally adjusted for major chronic comorbidities. Participants without depressive symptoms were defined as the reference group. HRs > 1 indicate increased risk of incident digestive disease. HRs, hazard ratios; CIs, confidence intervals; CESD-10, 10-item Center for Epidemiologic Studies Depression Scale.

**Fig. 5:**
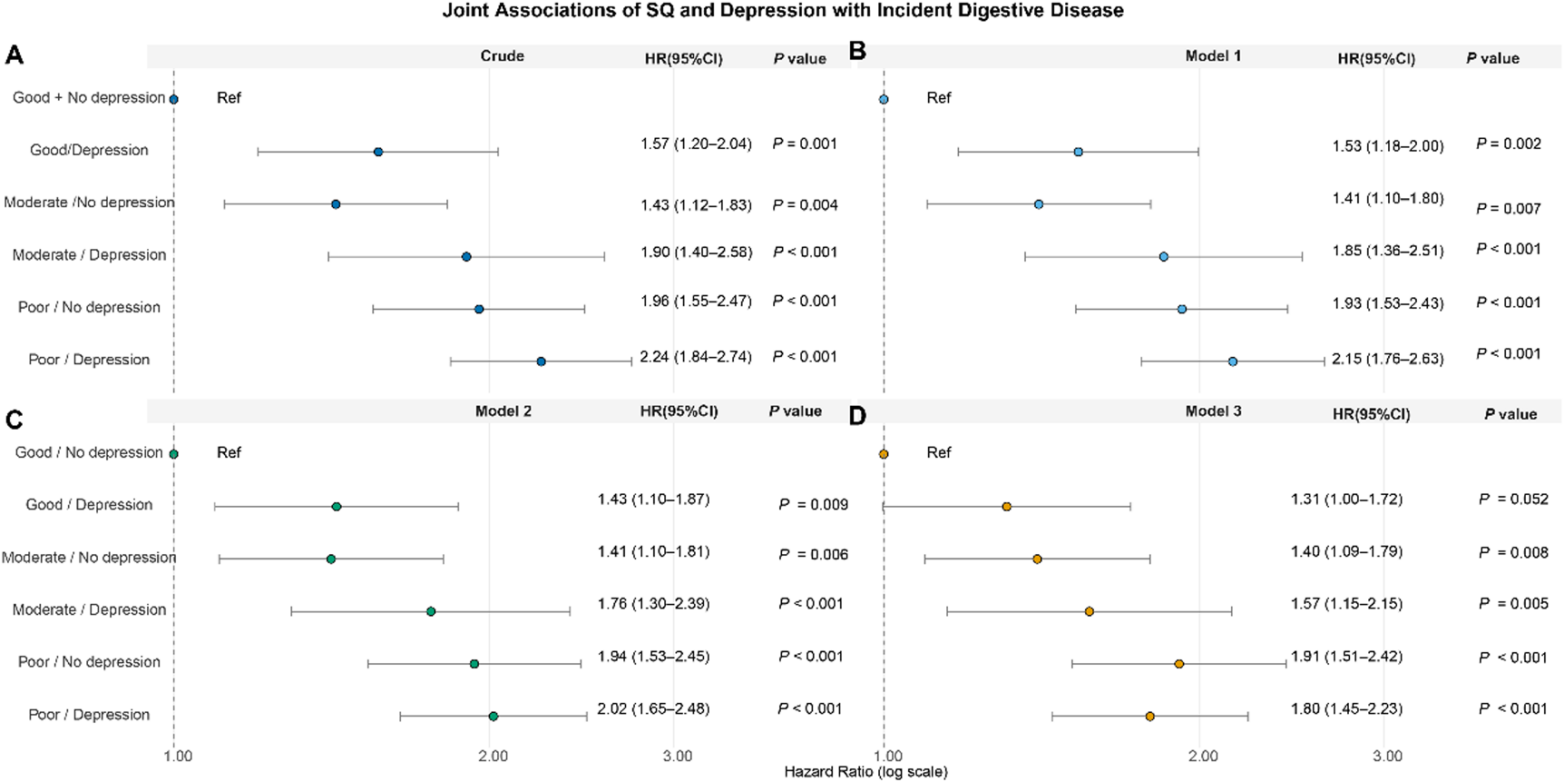
Joint associations of SQ and depressive symptoms with incident digestive disease. Forest plots illustrating the joint-category associations of SQ (good, moderate, poor) and depressive symptom status (CESD-10 ≥ 10 = Yes) on the risk of incident digestive disease across four hierarchical models. (**A**) Crude model; (**B**) Model 1 adjusted for age and sex; (**C**) Model 2 adjusted for sociodemographic factors; and (**D**) Model 3 fully adjusted for lifestyle factors and comorbidities. “Good SQ / No depressive symptoms” served as the reference group. HRs > 1 indicate increased risk compared with the reference. These estimates represent joint-category associations and were not interpreted as formal additive or multiplicative interaction analyses. SQ, sleep quality; CESD-10, 10-item Center for Epidemiologic Studies Depression Scale.

**Fig. 6:**
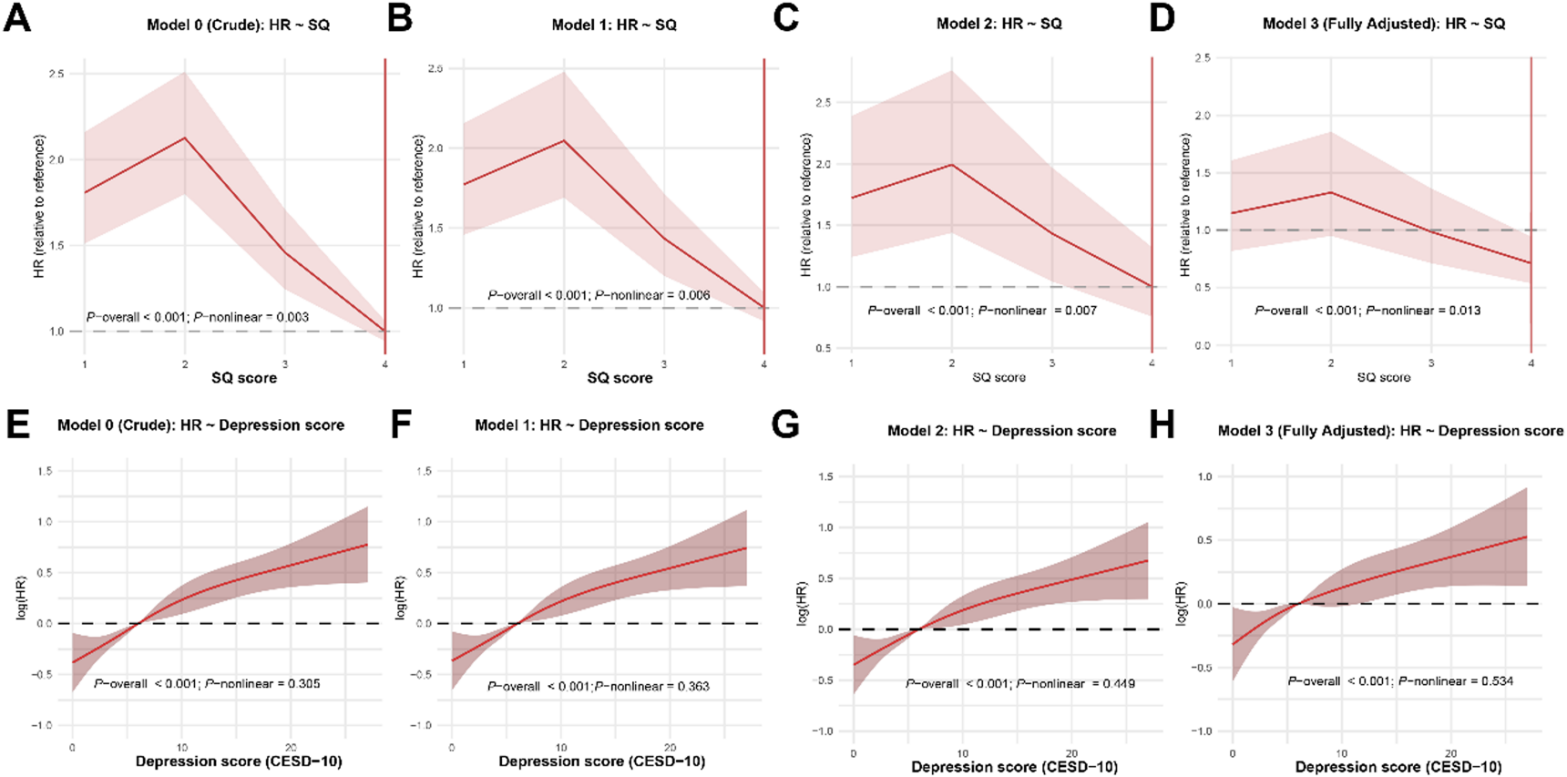
Nonlinear dose–response of SQ and depressive symptoms with digestive disease risk. (**A–D**) RCS curves for the association between SQ score and incident digestive disease across four models: (**A**) Crude; (**B**) Model 1 (age, sex); (**C**) Model 2 (Model 1 + sociodemographics); (**D**) Model 3 (Model 2 + lifestyle and comorbidities). Curves show hazard ratios (solid line) with 95% CIs (shaded band) relative to the reference. (**E–H**) RCS curves for the association between CESD-10 score (range 0–30) and incident digestive disease under the same four models. RCS, restricted cubic spline; SQ, sleep quality; CESD-10, 10-item Center for Epidemiologic Studies Depression Scale; CI, confidence interval; HR, hazard ratio.

Prospective analyses showed that poorer SQ was consistently associated with a higher risk of incident digestive disease (Fig. 3, Supplementary Table 4). Compared with good SQ, crude HRs were 1.46 (95% CI 1.19–1.78) for moderate SQ and 1.97 (95% CI 1.67–2.32) for poor SQ (both *P* < 0.001). After sequential adjustment, associations persisted with modest attenuation (Model 3: HR 1.36 [95% CI 1.11–1.68] for moderate and HR 1.67 [95% CI 1.39–2.01] for poor; both *P* ≤ 0.003). Depressive symptoms were also associated with increased risk of incident digestive disease (Fig. 4, Supplementary Table 5). The crude HR was 1.65 (95% CI 1.42–1.92; *P* < 0.001), and the association remained significant in the fully adjusted model (HR 1.33; 95% CI 1.13–1.57; *P* < 0.001). In the joint-category analysis (Fig. 5, Supplementary Table 6), compared with good SQ without depressive symptoms, elevated risks were observed for poor SQ without depressive symptoms (Model 3: HR 1.91; 95% CI 1.51–2.42; *P* < 0.001) and for poor SQ with depressive symptoms (HR 1.80; 95% CI 1.45–2.23; *P* < 0.001), whereas good SQ with depressive symptoms showed a borderline association (HR 1.31; 95% CI 1.00–1.72; *P* = 0.052). Dose–response analyses were concordant. SQ showed a significant inverse nonlinear association with incident digestive disease risk (*P*-overall < 0.001; *P* for nonlinearity = 0.003–0.013; Figs. 6A–D; Supplementary Table 7), with the steepest increase when SQ declined from 3 to 2. CESD-10 score showed a near-linear positive association with risk (*P*-overall < 0.001; *P* for nonlinearity > 0.30; Figs. 6E–H; Supplementary Table 8).

### Exploratory mediation analysis

**Fig. 7:**
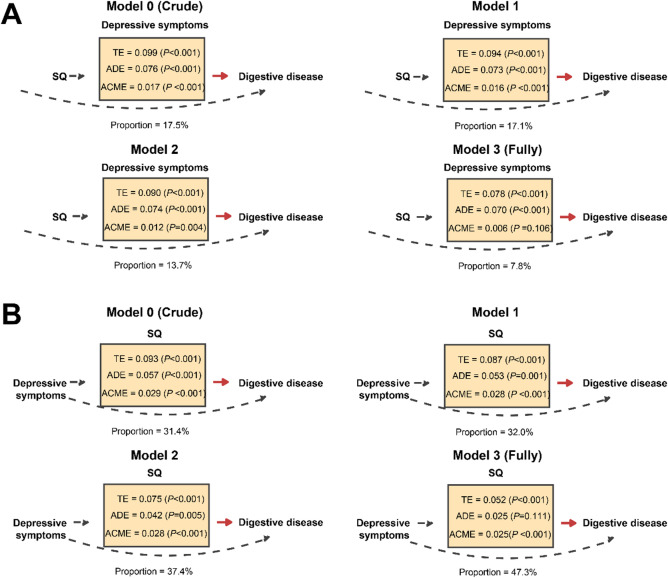
Mediation analysis between SQ, depressive symptoms, and incident digestive disease. (**A**) Mediation models evaluating depressive symptoms as the mediator in the association between SQ and incident digestive disease. (**B**) Mediation models evaluating SQ as the mediator in the association between depressive symptoms and incident digestive disease. Each panel represents results from four hierarchical models: Model 0 (crude), Model 1 (+ age, sex), Model 2 (+ sociodemographic variables), and Model 3 (+ lifestyle and comorbidities). Proportion, percentage of total effect mediated through the specified indirect association. SQ, sleep quality; TE, total effect; ADE, average direct effect; ACME, average causal mediation effect.

Exploratory mediation analyses were performed to evaluate potential model-based indirect associations among SQ, depressive symptoms, and incident digestive disease. When depressive symptoms were specified as the mediator of the SQ–incident digestive disease association, the indirect association was significant in the crude model (Model 0: ACME 0.017, 95% CI 0.007–0.028; *P* < 0.001) but was attenuated after full adjustment (Model 3: ACME 0.006, 95% CI − 0.002–0.015; *P* = 0.106). The estimated proportion mediated declined from 17.5% to 7.8% across models (Fig. 7A, Supplementary Table 9). In the alternative direction, when SQ was specified as the mediator of the depressive symptoms–incident digestive disease association, the indirect association was significant across models (Model 0: ACME 0.029, 95% CI 0.018–0.041; *P* < 0.001) and remained significant after full adjustment (Model 3: ACME 0.025, 95% CI 0.015–0.036; *P* < 0.001). The estimated proportion mediated ranged from 31.4% to 47.3% (Fig. 7B, Supplementary Table 10). These findings were interpreted as exploratory and assumption-dependent, rather than as confirmation of causal pathways.

###  Associations of longitudinal SQ-depressive symptom trajectory patterns with incident digestive disease risk

**Fig. 8:**
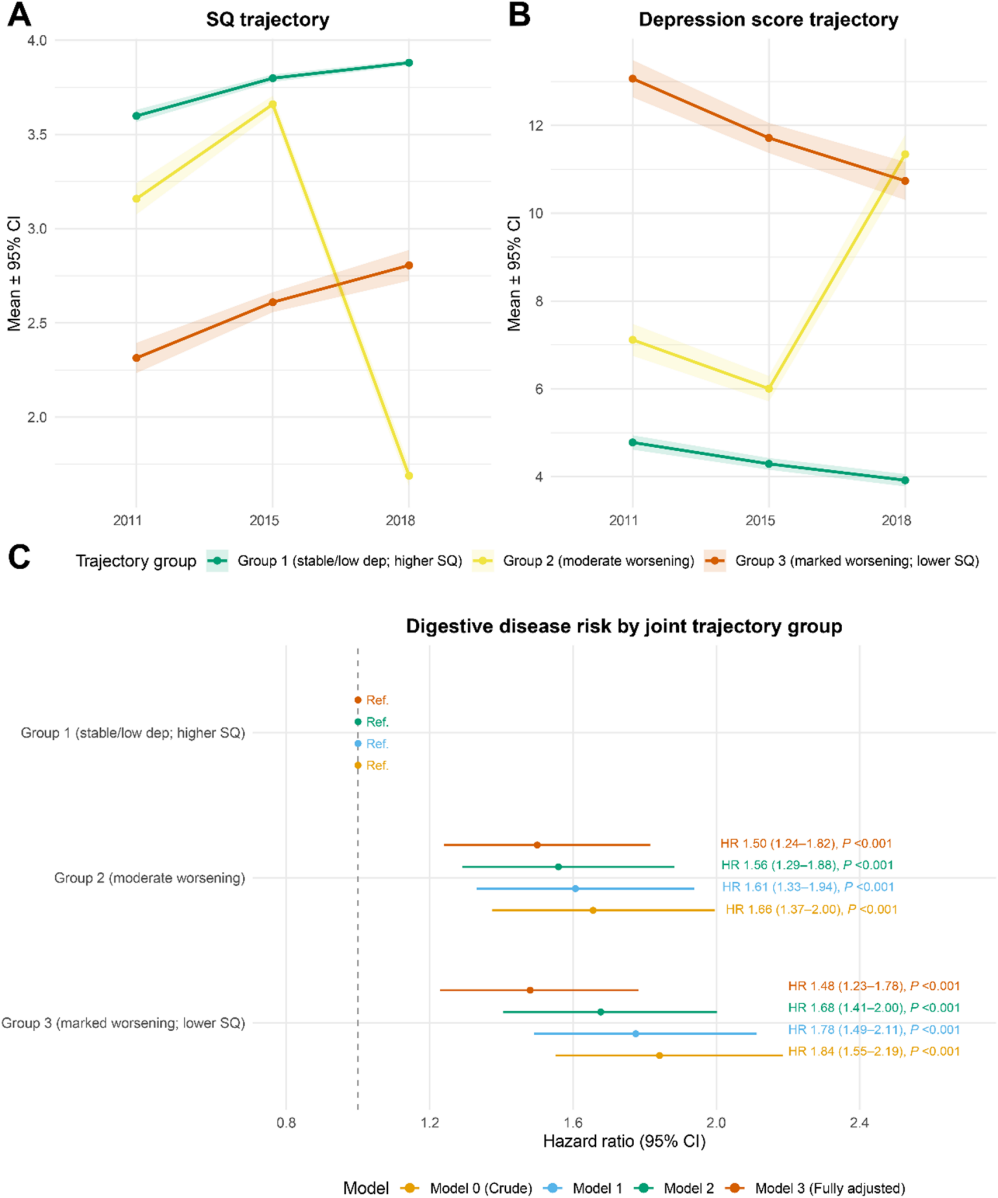
Longitudinal SQ–depressive symptom trajectory patterns in relation to incident digestive disease. (**A**) SQ trajectories and (**B**) depressive symptom score trajectories were classified into three distinct longitudinal trajectory patterns: Group 1 (stable/low depressive symptoms; higher SQ), Group 2 (moderate worsening), and Group 3 (marked worsening; lower SQ). (**C**) Forest plots showing hazard ratios (HRs) and 95% confidence intervals (CIs) for the risk of incident digestive disease across the three trajectory groups under four models: Model 0 (crude), Model 1 (age- and sex-adjusted), Model 2 (additionally adjusted for sociodemographic variables), and Model 3 (further adjusted for lifestyle and comorbidity factors). Group 1 served as the reference group. Groups 2 and 3 showed elevated risks of incident digestive disease relative to Group 1 across models. SQ, sleep quality; HR, hazard ratio; CI, confidence interval.

Trajectory-pattern analysis showed elevated risks of incident digestive disease across longitudinal SQ–depressive symptom pattern groups (Fig. 8, Supplementary Table 11). Using the group characterized by stable/low depressive symptoms and higher SQ as the reference group (Group 1), higher risks were observed for the moderate worsening group (Group 2) and the marked worsening/lower SQ group (Group 3). The crude HRs were 1.66 (95% CI 1.37–2.00) for Group 2 and 1.84 (95% CI 1.55–2.19) for Group 3 (both *P* < 0.001). These associations remained significant after full adjustment, with HRs of 1.50 (95% CI 1.24–1.82) and 1.48 (95% CI 1.23–1.78), respectively (both *P* < 0.001).

### Sensitivity analyses

The main findings were generally consistent across sensitivity analyses using alternative exposure definitions and analytic subgroups (Supplementary Fig. 2, Supplementary Tables 12–14). When SQ was modeled continuously, each 1-point decrease in SQ score was associated with a higher risk of incident digestive disease (HR = 1.21, 95% CI 1.14–1.30; *P* < 0.001). Similarly, each 1-SD increase in CESD-10 score (SD = 5.66) was associated with a higher risk (HR = 1.20, 95% CI 1.11–1.29; *P* < 0.001). When analyses were restricted to participants without baseline comorbidities, the association between poor SQ and incident digestive disease risk persisted (HR = 1.51, 95% CI 1.08–2.09; *P* = 0.015), whereas the association for depressive symptoms was attenuated and no longer statistically significant (HR = 1.13, 95% CI 0.80–1.58; *P* = 0.489). Given the overlap between the SQ measure and the CESD-10 restless-sleep item, additional sensitivity analyses were performed using modified CESD-9 after excluding this item. The association between SQ and modified CESD-9 was attenuated but remained significant (Spearman rho = − 0.324; *P* < 0.001; Supplementary Fig. 3). Across good, moderate, and poor SQ categories, mean modified CESD-9 scores increased from 4.85 to 6.90 and 9.10, and the prevalence of high modified CESD-9 burden increased from 18.1% to 31.6% and 48.8%, respectively (Supplementary Tables 15–16). Further analyses using modified CESD-9, including RCS models, high-burden Cox models, joint-category analyses, exploratory bidirectional mediation analyses, and joint trajectory analyses, showed attenuated but generally consistent patterns (Supplementary Figs. 4–8; Supplementary Tables 17–18). In the fully adjusted binary mediation models, the indirect association through high modified CESD-9 burden for the poorer SQ–incident digestive disease association was attenuated and no longer statistically significant (ACME = 0.0037; 95% CI − 0.0011–0.0089; *P* = 0.132; proportion mediated = 4.6%). In the alternative direction, the indirect association through poorer SQ for the high modified CESD-9 burden–incident digestive disease association remained evident after full adjustment (ACME = 0.0165; 95% CI 0.0100–0.0243; *P* < 0.001; proportion mediated = 40.2%; Supplementary Table 18). Proportional hazards assumption tests showed no evidence of violation for the main Cox models or sensitivity models (Supplementary Table 19).

Overall, the sensitivity analyses yielded broadly consistent results, while the modified CESD-9 analyses suggested that item overlap may have influenced the magnitude of the sleep–depressive symptom association.

## Discussion

In this national prospective cohort of middle-aged and older Chinese adults, poorer SQ and depressive symptoms were associated with higher subsequent risk of incident self-reported digestive disease. In separate models, both poorer SQ and depressive symptoms were associated with digestive disease risk. Joint-category analyses showed elevated risks among participants with poor SQ, both with and without depressive symptoms, relative to those with good SQ and no depressive symptoms. These findings should be interpreted descriptively and were not considered evidence of additive or multiplicative interaction. Trajectory-pattern analyses suggested elevated risk among participants with worsening longitudinal SQ–depressive symptom patterns. Exploratory mediation analyses suggested that the indirect association through depressive symptoms for the SQ–digestive disease association was attenuated after full adjustment, whereas the alternative indirect association through SQ for the depressive symptoms–digestive disease association remained evident. These findings should therefore be viewed as exploratory statistical decompositions under modelling assumptions, rather than as evidence for directional causal pathways. In sensitivity analyses, the overall patterns were generally consistent across alternative exposure definitions, restriction to participants without baseline comorbidities, and modified CESD-9 analyses excluding the restless-sleep item. Overall, these results support a potential sleep–mood–digestive health link, while causal and interventional interpretations remain limited by the observational design.

These results align with and extend observational evidence linking impaired sleep and depressive symptoms to gastrointestinal outcomes, while highlighting the value of a joint-category and trajectory-pattern perspective. Previous studies have reported bidirectional associations between sleep problems and gastro-oesophageal reflux symptoms, suggesting a reciprocal sleep–gut relationship^24,25^. Sleep disturbance has also been associated with functional dyspepsia, symptom burden, poorer quality of life, and abnormal bowel habits, supporting sleep as a relevant correlate of gastrointestinal function^26,27^. In parallel, mood-related factors have been linked to digestive disease risk: prior depression has been associated with higher subsequent inflammatory bowel disease risk and poorer prognosis, and clinically diagnosed depression has been reported to predict new-onset irritable bowel syndrome^13,28^. Lifestyle–genetic analyses further suggest that unhealthy behaviors, including insufficient sleep, may contribute to incident irritable bowel syndrome risk, especially among genetically susceptible individuals^29^. Evidence from shift-work and inflammatory bowel disease studies also supports the relevance of longitudinal sleep–mood–gut relationships, as rotating or night-shift work has been associated with incident irritable bowel syndrome partly through sleep and affective factors, whereas sleep problems in inflammatory bowel disease are common and related to disease activity and disability^30,31^. Together, these findings provide external support for the observed associations of SQ, depressive symptoms, and their longitudinal patterns with incident digestive disease, while remaining consistent with an observational rather than causal interpretation.

Biological plausibility may involve neuroendocrine, immune–inflammatory, microbial, and circadian pathways linking SQ, depressive symptoms, and digestive health. Stress-related activation of the hypothalamic–pituitary–adrenal axis and altered corticotropin-releasing hormone/glucocorticoid signalling may impair epithelial barrier integrity and contribute to visceral hypersensitivity, with evidence implicating mast-cell–dependent mechanisms in stress-related intestinal permeability changes^32^. Sleep disturbance and insomnia have been associated with systemic inflammatory activation, including inflammatory mediators such as IL-6 and CRP, which may affect mucosal immune responses and enteric neural function^33^. Sleep loss and psychological stress may also reshape the gut microbiota, alter tight-junction integrity, and influence microbial metabolites involved in permeability, enteroendocrine signalling, and brain–gut communication^34^. In addition, vagal afferent signalling may connect intestinal immune and metabolic states with central mood and pain networks, whereas circadian disruption can affect gastrointestinal motility, mucosal immunity, bile-acid metabolism, and microbiome rhythmicity^35,36^. These interconnected pathways offer hypotheses for the observed sleep–mood–digestive health associations, although the present observational findings cannot establish these mechanisms or directional pathways directly.

This study has several strengths. The use of a nationally representative longitudinal cohort enabled prospective assessment of SQ, depressive symptoms, and incident digestive disease in middle-aged and older adults. The analytic framework incorporated single-exposure Cox models, joint-category analyses, exploratory mediation analyses, trajectory-pattern analyses, and multiple sensitivity analyses, providing a comprehensive evaluation of the sleep–mood–digestive disease relationship. In addition, modified CESD-9 analyses after excluding the restless-sleep item helped address potential item overlap between SQ and depressive symptom assessment. Together, these approaches add population-based evidence on how SQ and depressive symptoms, individually and jointly, are associated with digestive disease risk.

Limitations warrant consideration. First, SQ and depressive symptoms were assessed using self-reported questionnaire-based measures rather than objective sleep assessments or a dedicated validated sleep instrument such as the Pittsburgh Sleep Quality Index, which may have introduced measurement error and limited characterization of specific sleep dimensions. The SQ measure also incorporated the CESD-10 restless-sleep item, creating potential item overlap with the CESD-10 depressive symptom scale. Sensitivity analyses using modified CESD-9 after removing this item showed attenuated but directionally consistent findings, suggesting that item overlap may have influenced the magnitude of the observed associations. Second, depressive symptoms were based on CESD-10 rather than clinical psychiatric diagnoses. Third, incident digestive disease was defined by self- or proxy-reported physician diagnosis of “stomach or other digestive disease,” representing a broad and heterogeneous outcome; disease-subtype-specific analyses were not available, and outcome misclassification cannot be excluded. Therefore, this outcome should be interpreted as reflecting heterogeneous digestive morbidity burden rather than mechanisms specific to any single digestive disease subtype. Fourth, the exploratory mediation analyses were assumption-dependent and should be viewed as exploratory statistical decompositions under modelling assumptions rather than evidence for directional causal pathways. Finally, residual confounding from unmeasured factors such as diet, medication use, health-care utilization, physical activity, and genetic susceptibility remains possible. Some adjusted covariate estimates, including inverse associations for hypertension and diabetes, should not be interpreted as protective effects and may reflect analytic cohort selection, competing covariate structures, residual bias, or outcome heterogeneity. Reverse causation cannot be fully excluded in this observational study.

These findings may inform observational risk assessment. SQ and depressive symptoms may be useful to consider together when evaluating digestive disease risk in middle-aged and older adults. Whether sleep- or mood-focused strategies can translate into lower digestive disease risk requires further investigation. Future research should use objective sleep measures, refined digestive disease phenotyping, external validation, and biological markers to clarify the sleep–mood–digestive health relationship and its potential modifiability.

## Conclusion

In conclusion, poor SQ and depressive symptoms were associated with higher subsequent risk of a broad, self-reported digestive disease outcome. Joint-category analyses showed elevated risk in poor SQ categories, while trajectory-pattern analyses suggested elevated risk among participants with worsening longitudinal SQ–depressive symptom patterns. Exploratory mediation analyses indicated potential model-based indirect associations under modelling assumptions. These findings support further investigation of the sleep–mood–digestive health relationship using objective sleep measures, refined digestive disease phenotyping, and external validation.

## Supplementary Information

Below is the link to the electronic supplementary material.

Supplementary Material 1

## Data Availability

The data used in this study are available from the CHARLS under controlled access. The analysis scripts used during the current study are available from the corresponding author on reasonable request.
