# Supplementary material for: Joint associations of sleep quality and depressive symptoms with incident digestive disease in a nationally representative Chinese cohort
Source: Sci Rep. 2026 Jun 5;16:25679. doi: 10.1038/s41598-026-56067-7 (PMC13478507; doi:10.1038/s41598-026-56067-7)
Supplement: Supplementary file 1 [file 41598_2026_56067_MOESM1_ESM.docx]

**Supplementary Materials**

content

[Supplementary Table 1. Baseline SQ score by depressive symptom status	3](#_Toc228453562)

[Supplementary Table 2. Baseline CESD-10 score by SQ category	3](#_Toc228453563)

[Supplementary Table 3. Multivariable linear regression models for the association between CESD-10 score and SQ	3](#_Toc228453564)

[Supplementary Table 4. Associations of baseline SQ and covariates with incident digestive disease risk in Cox proportional hazards models	8](#_Toc228453565)

[Supplementary Table 5. Associations of baseline depressive symptoms with incident digestive disease risk in Cox proportional hazards models	11](#_Toc228453566)

[Supplementary Table 6. Joint associations of SQ and baseline depressive symptoms with incident digestive disease risk in Cox proportional hazards models	14](#_Toc228453567)

[Supplementary Table 7. RCS–Cox models for the association between SQ score and incident digestive disease risk	20](#_Toc228453568)

[Supplementary Table 8. RCS–Cox models for the association between CESD-10 score and incident digestive disease risk	25](#_Toc228453569)

[Supplementary Table 9. Exploratory mediation analysis of the association between SQ and incident digestive disease with depressive symptoms as the mediator	29](#_Toc228453570)

[Supplementary Table 10. Exploratory mediation analysis of the association between depressive symptoms and incident digestive disease with SQ as the mediator	31](#_Toc228453571)

[Supplementary Table 11. Associations of joint trajectory groups and covariates with incident digestive disease risk across Cox proportional hazards models	32](#_Toc228453572)

[Supplementary Table 12. Baseline characteristics stratified by SQ group	36](#_Toc228453573)

[Supplementary Table 13. Baseline characteristics stratified by depressive symptom severity	39](#_Toc228453574)

[Supplementary Table 14. Baseline characteristics of participants without baseline comorbidities stratified by SQ category	41](#_Toc228453575)

[Supplementary Table 15. SQ score by high modified CESD-9 burden status at baseline	43](#_Toc228453576)

[Supplementary Table 16. Modified CESD-9 score by SQ category at baseline	44](#_Toc228453577)

[Supplementary Table 17. Association between modified CESD-9 score and SQ across multivariable linear regression models	44](#_Toc228453578)

[Supplementary Table 18. Exploratory bidirectional mediation analyses using modified CESD-9 after excluding the restless-sleep item	49](#_Toc228453579)

[Supplementary Table 19. Proportional hazards assumption tests for key Cox models	51](#_Toc228453580)

[Supplementary Figure 1. Nonlinear association between CESD-10 score and SQ.	53](#_Toc228453581)

[Supplementary Figure 2. Sensitivity analyses for the associations of SQ and depressive symptoms with incident digestive disease.	53](#_Toc228453582)

[Supplementary Figure 3. Association between SQ and modified CESD-9 after excluding the restless-sleep item.	54](#_Toc228453583)

[Supplementary Figure 4. Nonlinear association between modified CESD-9 and SQ.	55](#_Toc228453584)

[Supplementary Figure 5. Associations of high modified CESD-9 burden with incident digestive disease.	56](#_Toc228453585)

[Supplementary Figure 6. Joint associations of sleep quality and high modified CESD-9 burden with incident digestive disease.	56](#_Toc228453586)

[Supplementary Figure 7. Dose–response association between modified CESD-9 and incident digestive disease.	57](#_Toc228453587)

[Supplementary Figure 8. Modified CESD-9 joint trajectory sensitivity analysis in relation to incident digestive disease.	58](#_Toc228453588)

# Supplementary Table 1. Baseline SQ score by depressive symptom status

| **Depressive symptoms (CESD-10 ≥ 10)** | **n** | **Mean SQ** | **SD SQ** | **Median SQ** | **Q1** | **Q3** |
| --- | --- | --- | --- | --- | --- | --- |
| No | 2,693 | 3.51 | 0.88 | 4 | 3 | 4 |
| Yes | 1,018 | 2.56 | 1.17 | 2 | 1 | 4 |

*Notes*: SQ, sleep quality; CESD-10, 10-item Center for Epidemiologic Studies Depression Scale; SD, standard deviation; Q1/Q3, first/third quartile. Depressive symptoms were defined as CESD-10 score ≥10.

# Supplementary Table 2. Baseline CESD-10 score by SQ category

| **SQ category** | **n** | **Mean CESD-10** | **SD** | **Median CESD-10** | **Q1** | **Q3** | **CESD-10 (≥10)** |
| --- | --- | --- | --- | --- | --- | --- | --- |
| Good | 2,227 | 4.85 | 4.41 | 4 | 1 | 7 | 14.1% |
| Moderate | 611 | 7.90 | 4.83 | 7 | 4 | 11 | 31.6% |
| Poor | 873 | 11.59 | 6.06 | 11 | 7 | 15 | 58.4% |

Notes: SQ, sleep quality; CESD-10, 10-item Center for Epidemiologic Studies Depression Scale; SD, standard deviation; Q1/Q3, first/third quartile. Depressive symptoms were defined as CESD-10 score ≥10.

# Supplementary Table 3. Multivariable linear regression models for the association between CESD-10 score and SQ

| **Model** | **Variable** | **Term** | **β (Estimate)** | **SE** | **95% CI (Lower, Upper)** | ***P* value** |
| --- | --- | --- | --- | --- | --- | --- |
| **Crude (unadjusted)** | Intercept | Intercept | 3.99 | 0.04 | 3.91 – 4.06 | <0.001 |
|  | CESD-10 score (linear term) | CESD-10 score | −0.13 | 0.02 | −0.16 – −0.10 | <0.001 |
|  | CESD-10 score (nonlinear term 1) | CESD-10 score | 0.12 | 0.09 | −0.06 – 0.31 | 0.191 |
|  | CESD-10 score (nonlinear term 2) | CESD-10 score | −0.22 | 0.22 | −0.65 – 0.21 | 0.312 |
| **Model 1 (Age, Sex)** | Intercept | Intercept | 4.04 | 0.11 | 3.82 – 4.27 | <0.001 |
|  | CESD-10 score (linear term) | CESD-10 score | −0.13 | 0.02 | −0.16 – −0.09 | <0.001 |
|  | CESD-10 score (nonlinear term 1) | CESD-10 score | 0.12 | 0.09 | −0.06 – 0.31 | 0.193 |
|  | CESD-10 score (nonlinear term 2) | CESD-10 score | −0.22 | 0.22 | −0.65 – 0.21 | 0.312 |
|  | Sex (Female vs Male) | Sex=Female | −0.13 | 0.05 | −0.23 – −0.04 | 0.009 |
|  | Age (per year) | Age | −0.01 | 0.00 | −0.02 – −0.00 | 0.008 |
| **Model 2 (+ socio-demographic)** | Intercept | Intercept | 3.96 | 0.13 | 3.70 – 4.22 | <0.001 |
|  | CESD-10 score (linear term) | CESD-10 score | −0.12 | 0.02 | −0.15 – −0.09 | <0.001 |
|  | CESD-10 score (nonlinear term 1) | CESD-10 score | 0.12 | 0.09 | −0.06 – 0.31 | 0.193 |
|  | CESD-10 score (nonlinear term 2) | CESD-10 score | −0.22 | 0.22 | −0.65 – 0.21 | 0.312 |
|  | Sex (Female vs Male) | Sex=Female | −0.12 | 0.05 | −0.23 – −0.03 | 0.012 |
|  | Age (per year) | Age | −0.01 | 0.00 | −0.02 – −0.00 | 0.010 |
|  | Education (≥High school vs < High school) | High | 0.15 | 0.06 | 0.04 – 0.26 | 0.007 |
|  | Marital status (Unmarried vs Married) | Marry =Unmarried | −0.09 | 0.05 | −0.19 – 0.01 | 0.081 |
|  | Residence (Urban vs Rural) | Live place=Urban | 0.05 | 0.05 | −0.05 – 0.15 | 0.327 |
| **Model 3 (+ lifestyle & comorbidities)** | Intercept | Intercept | 4.00 | 0.15 | 3.71 – 4.29 | <0.001 |
|  | CESD-10 score (linear term) | CESD-10 score | −0.12 | 0.02 | −0.15 – −0.09 | <0.001 |
|  | CESD-10 score (nonlinear term 1) | CESD-10 score | 0.12 | 0.09 | −0.06 – 0.31 | 0.193 |
|  | CESD-10 score (nonlinear term 2) | CESD-10 score | −0.22 | 0.22 | −0.65 – 0.21 | 0.312 |
|  | Sex (Female vs Male) | Sex=Female | −0.13 | 0.05 | −0.23 – −0.03 | 0.011 |
|  | Age (per year) | Age | −0.01 | 0.00 | −0.02 – −0.00 | 0.012 |
|  | BMI category (Overweight vs Normal) | BMI =Overweight | −0.05 | 0.05 | −0.15 – 0.05 | 0.329 |
|  | BMI category (Obesity vs Normal) | BMI =Obese | −0.09 | 0.07 | −0.23 – 0.05 | 0.198 |
|  | Smoking status (Current vs Never) | Smoke status=Current | −0.07 | 0.06 | −0.19 – 0.04 | 0.198 |
|  | Drinking status (Current vs Never) | Drink status=Current | 0.10 | 0.06 | −0.02 – 0.22 | 0.097 |
|  | Hypertension (Yes vs No) | Hypertension=Yes | −0.10 | 0.05 | −0.20 – 0.00 | 0.047 |
|  | Heart disease (Yes vs No) | Heart disease=Yes | −0.14 | 0.06 | −0.25 – −0.03 | 0.014 |
|  | Lung disease (Yes vs No) | Lung disease=Yes | −0.09 | 0.06 | −0.21 – 0.02 | 0.114 |
|  | Arthritis (Yes vs No) | Arthritis=Yes | −0.12 | 0.05 | −0.22 – −0.02 | 0.019 |
|  | Pain (Yes vs No) | Pain=Yes | −0.21 | 0.05 | −0.31 – −0.11 | <0.001 |

*Notes*: Results are from ordinary least-squares regression models with SQ score as the dependent variable. CESD-10 score was modeled using restricted cubic splines with four knots at 0, 4, 8, and 18. Individual spline coefficients should not be interpreted separately; the overall and nonlinear associations should be evaluated using joint tests. Model 0 was unadjusted. Model 1 adjusted for age and sex. Model 2 further adjusted for marital status, education, and residence. Model 3 additionally adjusted for lifestyle factors and chronic comorbidities. Selected covariate estimates are shown. Because higher SQ indicates better sleep, a negative β indicates an association with lower SQ, that is, poorer SQ. SE, standard error; CI, confidence interval; SQ, sleep quality; CESD-10, 10-item Center for Epidemiologic Studies Depression Scale; BMI, body mass index.

# Supplementary Table 4. Associations of baseline SQ and covariates with incident digestive disease risk in Cox proportional hazards models

| **Model** | **Variable** | **HR** | **95% CI** | ***P* value** |
| --- | --- | --- | --- | --- |
| **Model 0 (Crude)** | SQ – Moderate vs Good | 1.46 | 1.19–1.78 | <0.001 |
|  | SQ – Poor vs Good | 1.97 | 1.67–2.32 | <0.001 |
| **Model 1 (Age, Sex)** | SQ – Moderate vs Good | 1.43 | 1.17–1.75 | <0.001 |
|  | SQ – Poor vs Good | 1.91 | 1.62–2.26 | <0.001 |
|  | Age (per year) | 1.01 | 1.00–1.02 | 0.106 |
|  | Sex (Male vs Female) | 0.83 | 0.71–0.96 | 0.012 |
| **Model 2 (+ socio-demographic)** | SQ – Moderate vs Good | 1.43 | 1.17–1.75 | <0.001 |
|  | SQ – Poor vs Good | 1.86 | 1.57–2.21 | <0.001 |
|  | Age (per year) | 1.00 | 0.99–1.01 | 0.752 |
|  | Sex (Male vs Female) | 0.86 | 0.68–1.09 | 0.211 |
|  | Education – High school or above vs Primary or less | 0.82 | 0.61–1.10 | 0.186 |
|  | Marital status – Other vs Married | 1.24 | 0.97–1.58 | 0.085 |
|  | Residence – Rural vs Urban | 1.00 | 0.85–1.17 | 0.981 |
|  | BMI – Underweight vs Normal | 1.48 | 1.11–1.98 | 0.008 |
|  | BMI – Overweight vs Normal | 0.99 | 0.83–1.18 | 0.878 |
|  | BMI – Obesity vs Normal | 0.83 | 0.65–1.07 | 0.135 |
|  | Smoking – Ever vs Current | 0.90 | 0.67–1.21 | 0.508 |
|  | Drinking – Ever vs Current | 1.54 | 1.18–2.01 | 0.001 |
| **Model 3 (+ comorbidities & depressive symptoms)** | SQ – Moderate vs Good | 1.36 | 1.11–1.67 | 0.003 |
|  | SQ – Poor vs Good | 1.67 | 1.39–2.01 | <0.001 |
|  | Age (per year) | 1.00 | 0.99–1.01 | 0.537 |
|  | Sex (Male vs Female) | 0.91 | 0.72–1.14 | 0.427 |
|  | Education – High school or above vs Primary or less | 0.82 | 0.61–1.10 | 0.189 |
|  | Marital status – Other vs Married | 1.27 | 0.99–1.64 | 0.055 |
|  | Residence – Rural vs Urban | 0.96 | 0.81–1.13 | 0.610 |
|  | BMI – Underweight vs Normal | 1.35 | 1.01–1.80 | 0.042 |
|  | BMI – Overweight vs Normal | 0.98 | 0.83–1.16 | 0.856 |
|  | BMI – Obesity vs Normal | 0.84 | 0.65–1.10 | 0.188 |
|  | Smoking – Ever vs Current | 0.87 | 0.64–1.19 | 0.359 |
|  | Drinking – Ever vs Current | 1.53 | 1.17–2.00 | 0.002 |
|  | Hypertension (Yes vs No) | 0.75 | 0.62–0.91 | 0.003 |
|  | Diabetes (Yes vs No) | 0.67 | 0.46–0.97 | 0.034 |
|  | Dyslipidaemia (Yes vs No) | 1.28 | 0.97–1.70 | 0.078 |
|  | Heart disease (Yes vs No) | 1.45 | 1.14–1.84 | 0.002 |
|  | Liver disease (Yes vs No) | 1.62 | 1.09–2.40 | 0.016 |
|  | Kidney disease (Yes vs No) | 1.46 | 1.07–2.00 | 0.018 |
|  | Arthritis (Yes vs No) | 1.34 | 1.14–1.57 | <0.001 |
|  | Pain (Yes vs No) | 1.18 | 0.99–1.40 | 0.064 |
|  | Depressive symptoms (CESD-10 ≥10 vs <10) | 1.11 | 0.92–1.34 | 0.273 |

*Notes*: HRs and 95% CIs were estimated using Cox proportional hazards models with follow-up time in months. Good SQ served as the reference category. Model 0 was unadjusted. Model 1 adjusted for age and sex. Model 2 further adjusted for education, marital status, residence, BMI, smoking status, and drinking status. Model 3 additionally adjusted for chronic comorbidities and depressive symptoms. HR >1 indicates a higher risk of incident digestive disease. SQ, sleep quality; HR, hazard ratio; CI, confidence interval; BMI, body mass index; CESD-10, 10-item Center for Epidemiologic Studies Depression Scale.

# Supplementary Table 5. Associations of baseline depressive symptoms with incident digestive disease risk in Cox proportional hazards models

| **Model** | **Variable** | **HR** | **95% CI** | ***P* value** |
| --- | --- | --- | --- | --- |
| **Model 0 (Crude)** | **Depressive symptoms (Yes vs No)** | 1.65 | 1.42–1.92 | <0.001 |
| **Model 1 (Age, Sex)** | **Depressive symptoms (Yes vs No)** | 1.60 | 1.37–1.86 | <0.001 |
|  | Age (per year) | 1.01 | 1–1.02 | 0.136 |
|  | Sex: Male vs Female | 0.81 | 0.70–0.94 | 0.006 |
| **Model 2 (+ socio-demographic)** | **Depressive symptoms (Yes vs No)** | 1.50 | 1.28–1.75 | <0.001 |
|  | Age (per year) | 1.00 | 0.99–1.01 | 0.794 |
|  | Sex: Male vs Female | 0.84 | 0.67–1.06 | 0.151 |
|  | Education: High school or above | 0.82 | 0.61–1.10 | 0.176 |
|  | Education: Illiterate | 1.05 | 0.84–1.30 | 0.684 |
|  | Education: Literate | 1.06 | 0.85–1.32 | 0.610 |
|  | Education: Middle school | 0.88 | 0.70–1.10 | 0.265 |
|  | Residence: Rural vs Urban | 0.96 | 0.81–1.13 | 0.625 |
|  | Marital status: Other vs Married | 1.22 | 0.96–1.57 | 0.106 |
|  | BMI: Underweight (<18.5) vs Normal | 1.48 | 1.11–1.98 | 0.008 |
|  | BMI: Overweight (24.0–27.9) vs Normal | 0.97 | 0.82–1.16 | 0.766 |
|  | BMI: Obesity (≥28.0) vs Normal | 0.86 | 0.67–1.09 | 0.212 |
|  | Smoking: Ever vs Current | 0.89 | 0.66–1.21 | 0.450 |
|  | Smoking: Never vs Current | 0.97 | 0.78–1.21 | 0.810 |
|  | Drinking: Ever vs Current | 1.48 | 1.13–1.94 | 0.004 |
|  | Drinking: Never vs Current | 1.05 | 0.86–1.27 | 0.652 |
| **Model 3 (+ lifestyle & comorbidities)** | **Depressive symptoms (Yes vs No)** | 1.33 | 1.13–1.57 | <0.001 |
|  | Age (per year) | 1.00 | 0.99–1.01 | 0.541 |
|  | Sex: Male vs Female | 0.88 | 0.70–1.11 | 0.290 |
|  | Education: High school or above | 0.81 | 0.61–1.09 | 0.171 |
|  | Education: Illiterate | 1.07 | 0.86–1.33 | 0.554 |
|  | Education: Literate | 1.02 | 0.81–1.27 | 0.882 |
|  | Education: Middle school | 0.89 | 0.71–1.12 | 0.305 |
|  | Residence: Rural vs Urban | 0.93 | 0.79–1.10 | 0.425 |
|  | Marital status: Other vs Married | 1.28 | 1.00–1.64 | 0.051 |
|  | BMI: Underweight (<18.5) vs Normal | 1.40 | 1.05–1.87 | 0.023 |
|  | BMI: Overweight (24.0–27.9) vs Normal | 0.97 | 0.81–1.15 | 0.731 |
|  | BMI: Obesity (≥28.0) vs Normal | 0.85 | 0.66–1.10 | 0.217 |
|  | Smoking: Ever vs Current | 0.86 | 0.63–1.17 | 0.335 |
|  | Smoking: Never vs Current | 0.99 | 0.79–1.24 | 0.938 |
|  | Drinking: Ever vs Current | 1.48 | 1.13–1.95 | 0.004 |
|  | Drinking: Never vs Current | 1.06 | 0.87–1.28 | 0.571 |
|  | Hypertension: Yes vs No | 0.77 | 0.63–0.93 | 0.007 |
|  | Diabetes: Yes vs No | 0.67 | 0.46–0.98 | 0.038 |
|  | Dyslipidaemia: Yes vs No | 1.25 | 0.95–1.64 | 0.109 |
|  | Heart disease: Yes vs No | 1.46 | 1.15–1.86 | 0.002 |
|  | Liver disease: Yes vs No | 1.64 | 1.11–2.42 | 0.013 |
|  | Lung disease: Yes vs No | 1.16 | 0.88–1.52 | 0.306 |
|  | Stroke: Yes vs No | 0.94 | 0.54–1.66 | 0.841 |
|  | Kidney disease: Yes vs No | 1.48 | 1.08–2.02 | 0.014 |
|  | Asthma: Yes vs No | 1.09 | 0.74–1.60 | 0.662 |
|  | Arthritis: Yes vs No | 1.35 | 1.15–1.59 | <0.001 |
|  | Pain: Yes vs No | 1.22 | 1.03–1.45 | 0.023 |
|  | Memory disorder: Yes vs No | 0.27 | 0.07–1.10 | 0.069 |
|  | Psychological disorder: Yes vs No | 1.34 | 0.59–3.01 | 0.485 |

*Notes*: HRs and 95% CIs were estimated using Cox proportional hazards models with follow-up time in months. Participants without depressive symptoms served as the reference group. Depressive symptoms were defined as CESD-10 score ≥10. Model 0 was unadjusted. Model 1 adjusted for age and sex. Model 2 further adjusted for education level, residence, marital status, BMI, smoking status, and drinking status. Model 3 additionally adjusted for chronic comorbidities. HR >1 indicates a higher risk of incident digestive disease. HR, hazard ratio; CI, confidence interval; CESD-10, 10-item Center for Epidemiologic Studies Depression Scale; BMI, body mass index.

# Supplementary Table 6. Joint associations of SQ and baseline depressive symptoms with incident digestive disease risk in Cox proportional hazards models

| **Model** | **Variable** | **HR** | **95% CI** | ***P* value** |
| --- | --- | --- | --- | --- |
| **Model 0 (Crude)** | Good SQ / No depressive symptoms (Ref.) |  |  |  |
|  | Good SQ / Depressive symptoms | 1.57 | 1.20–2.04 | <0.001 |
|  | Moderate SQ / Depressive symptoms | 1.90 | 1.40–2.58 | <0.001 |
|  | Moderate SQ / No depressive symptoms | 1.43 | 1.12–1.83 | 0.004 |
|  | Poor SQ / Depressive symptoms | 2.24 | 1.84–2.74 | <0.001 |
|  | Poor SQ / No depressive symptoms | 1.96 | 1.55–2.47 | <0.001 |
| **Model 1 (Age, Sex)** | Good SQ / Depressive symptoms | 1.53 | 1.18–2.00 | 0.002 |
|  | Moderate SQ / Depressive symptoms | 1.85 | 1.36–2.51 | <0.001 |
|  | Moderate SQ / No depressive symptoms | 1.41 | 1.10–1.80 | 0.007 |
|  | Poor SQ / Depressive symptoms | 2.15 | 1.76–2.63 | <0.001 |
|  | Poor SQ / No depressive symptoms | 1.93 | 1.53–2.43 | <0.001 |
|  | Age (per year) | 1.01 | 1.00–1.02 | 0.149 |
|  | Sex: Male vs Female | 0.84 | 0.72–0.98 | 0.023 |
| **Model 2 (+ socio-demographic)** | Good SQ / Depressive symptoms | 1.43 | 1.10–1.87 | 0.009 |
|  | Moderate SQ / Depressive symptoms | 1.76 | 1.30–2.39 | <0.001 |
|  | Moderate SQ / No depressive symptoms | 1.41 | 1.10–1.81 | 0.006 |
|  | Poor SQ / Depressive symptoms | 2.02 | 1.65–2.48 | <0.001 |
|  | Poor SQ / No depressive symptoms | 1.94 | 1.53–2.45 | <0.001 |
|  | Age (per year) | 1.00 | 0.99–1.01 | 0.762 |
|  | BMI: Obesity (≥28.0) vs Normal | 0.84 | 0.65–1.07 | 0.156 |
|  | BMI: Overweight (24.0–27.9) vs Normal | 0.99 | 0.84–1.18 | 0.942 |
|  | BMI: Underweight (<18.5) vs Normal | 1.46 | 1.09–1.95 | 0.011 |
|  | Drinking: Ever vs Current | 1.50 | 1.15–1.96 | 0.003 |
|  | Drinking: Never vs Current | 1.05 | 0.86–1.28 | 0.626 |
|  | Education: High school or above | 0.84 | 0.62–1.12 | 0.234 |
|  | Education: Illiterate | 1.05 | 0.84–1.30 | 0.679 |
|  | Education: Literate | 1.07 | 0.86–1.34 | 0.528 |
|  | Education: Middle school | 0.87 | 0.70–1.10 | 0.250 |
|  | Marital status: Other vs Married | 1.23 | 0.96–1.57 | 0.103 |
|  | Residence: Rural vs Urban | 0.99 | 0.84–1.17 | 0.891 |
|  | Sex: Male vs Female | 0.88 | 0.69–1.10 | 0.263 |
|  | Smoking: Ever vs Current | 0.90 | 0.66–1.22 | 0.495 |
|  | Smoking: Never vs Current | 0.98 | 0.78–1.22 | 0.838 |
| **Model 3 (+ lifestyle & comorbidities)** | Good SQ / Depressive symptoms | 1.31 | 1.00–1.72 | 0.052 |
|  | Moderate SQ / Depressive symptoms | 1.57 | 1.15–2.15 | 0.005 |
|  | Moderate SQ / No depressive symptoms | 1.40 | 1.09–1.79 | 0.008 |
|  | Poor SQ / Depressive symptoms | 1.80 | 1.45–2.23 | <0.001 |
|  | Poor SQ / No depressive symptoms | 1.91 | 1.51–2.42 | <0.001 |
|  | Age (per year) | 1.00 | 0.99–1.01 | 0.551 |
|  | Arthritis: Yes vs No | 1.34 | 1.14–1.57 | <0.001 |
|  | Asthma: Yes vs No | 1.11 | 0.76–1.63 | 0.596 |
|  | BMI: Obesity (≥28.0) vs Normal | 0.84 | 0.65–1.09 | 0.189 |
|  | BMI: Overweight (24.0–27.9) vs Normal | 0.99 | 0.83–1.17 | 0.868 |
|  | BMI: Underweight (<18.5) vs Normal | 1.37 | 1.02–1.83 | 0.036 |
|  | Diabetes: Yes vs No | 0.66 | 0.46–0.96 | 0.030 |
|  | Drinking: Ever vs Current | 1.53 | 1.17–2.01 | 0.002 |
|  | Drinking: Never vs Current | 1.06 | 0.87–1.29 | 0.549 |
|  | Dyslipidaemia: Yes vs No | 1.28 | 0.98–1.69 | 0.074 |
|  | Education: High school or above | 0.83 | 0.62–1.11 | 0.213 |
|  | Education: Illiterate | 1.07 | 0.86–1.33 | 0.540 |
|  | Education: Literate | 1.03 | 0.83–1.29 | 0.768 |
|  | Education: Middle school | 0.89 | 0.70–1.11 | 0.299 |
|  | Heart disease: Yes vs No | 1.46 | 1.15–1.86 | 0.002 |
|  | Hypertension: Yes vs No | 0.74 | 0.61–0.90 | 0.003 |
|  | Kidney disease: Yes vs No | 1.46 | 1.07–2.00 | 0.017 |
|  | Liver disease: Yes vs No | 1.62 | 1.10–2.40 | 0.015 |
|  | Lung disease: Yes vs No | 1.14 | 0.87–1.51 | 0.343 |
|  | Marital status: Other vs Married | 1.27 | 1.00–1.63 | 0.055 |
|  | Memory disorder: Yes vs No | 0.27 | 0.07–1.10 | 0.067 |
|  | Pain: Yes vs No | 1.17 | 0.99–1.39 | 0.065 |
|  | Psychological disorder: Yes vs No | 1.32 | 0.58–2.97 | 0.510 |
|  | Residence: Rural vs Urban | 0.96 | 0.81–1.14 | 0.643 |
|  | Sex: Male vs Female | 0.91 | 0.72–1.15 | 0.432 |
|  | Smoking: Ever vs Current | 0.87 | 0.64–1.18 | 0.369 |
|  | Smoking: Never vs Current | 1.00 | 0.80–1.25 | 0.981 |
|  | Stroke: Yes vs No | 0.94 | 0.53–1.64 | 0.819 |

***Notes*:** Good SQ without depressive symptoms served as the reference group. Depressive symptoms were defined as CESD-10 score ≥10. Model 0 was unadjusted. Model 1 adjusted for age and sex. Model 2 further adjusted for education level, residence, marital status, BMI, smoking status, and drinking status. Model 3 included all Model 2 covariates and additionally adjusted for chronic comorbidities. These results represent joint-category associations and were not interpreted as evidence of additive or multiplicative interaction. HR >1 indicates a higher risk of incident digestive disease. SQ, sleep quality; CESD-10, 10-item Center for Epidemiologic Studies Depression Scale; HR, hazard ratio; CI, confidence interval; BMI, body mass index.

# Supplementary Table 7. RCS–Cox models for the association between SQ score and incident digestive disease risk

| **Model** | **Variable** | **Term** | **β (Estimate)** | **SE** | **95% CI (Lower, Upper)** | **P value** |
| --- | --- | --- | --- | --- | --- | --- |
| **Model 0 (Crude)** | SQ score (RCS) | (linear term) | 0.306 | 0.187 | −0.060 – 0.672 | 0.101 |
|  | SQ score (RCS) | (nonlinear term 1) | −1.290 | 0.609 | −2.486 – −0.097 | 0.034 |
|  | SQ score (RCS) | (nonlinear term 2) | 2.900 | 1.750 | −0.532 – 6.337 | 0.098 |
| **Model 1 (Age, Sex)** | SQ score (RCS) | (linear term) | 0.276 | 0.187 | −0.090 – 0.643 | 0.140 |
|  | SQ score (RCS) | (nonlinear term 1) | −1.190 | 0.610 | −2.390 – 0.003 | 0.050 |
|  | SQ score (RCS) | (nonlinear term 2) | 2.670 | 1.750 | −0.765 – 6.112 | 0.128 |
|  | Age (per year) | Age | 0.007 | 0.005 | −0.002 – 0.016 | 0.109 |
|  | Sex | Sex = Male (vs Female) | −0.186 | 0.076 | −0.334 – −0.037 | 0.014 |
| **Model 2 (+ socio-demographic)** | SQ score (RCS) | (linear term) | 0.271 | 0.187 | −0.096 – 0.637 | 0.148 |
|  | SQ score (RCS) | (nonlinear term 1) | −1.120 | 0.611 | −2.322 – 0.075 | 0.066 |
|  | SQ score (RCS) | (nonlinear term 2) | 2.460 | 1.760 | −0.983 – 5.905 | 0.161 |
|  | Age (per year) | Age | −0.002 | 0.005 | −0.012 – 0.008 | 0.741 |
|  | BMI | Obesity (≥28.0) vs Normal | −0.186 | 0.125 | −0.432 – 0.060 | 0.138 |
|  | BMI | Overweight (24.0–27.9) vs Normal | −0.013 | 0.088 | −0.185 – 0.159 | 0.883 |
|  | BMI | Underweight (<18.5) vs Normal | 0.394 | 0.147 | 0.105 – 0.683 | 0.007 |
|  | Education | High school or above vs lower | −0.197 | 0.150 | −0.491 – 0.097 | 0.189 |
|  | Education | Illiterate vs lower | 0.054 | 0.110 | −0.162 – 0.270 | 0.625 |
|  | Education | Literate vs lower | 0.081 | 0.113 | −0.141 – 0.304 | 0.472 |
|  | Education | Middle school vs lower | −0.141 | 0.116 | −0.369 – 0.087 | 0.225 |
|  | Sex | Sex = Male (vs Female) | −0.143 | 0.118 | −0.375 – 0.089 | 0.226 |
|  | Drinking status | Ever vs Current | 0.432 | 0.137 | 0.164 – 0.699 | 0.002 |
|  | Drinking status | Never vs Current | 0.050 | 0.100 | −0.145 – 0.245 | 0.615 |
|  | Residence | Rural vs Urban | 0.005 | 0.084 | −0.159 – 0.169 | 0.953 |
|  | Marital status | Other vs Married | 0.218 | 0.125 | −0.028 – 0.463 | 0.082 |
|  | Smoking status | Ever vs Current | −0.104 | 0.155 | −0.408 – 0.201 | 0.505 |
|  | Smoking status | Never vs Current | −0.032 | 0.112 | −0.252 – 0.188 | 0.774 |
| **Model 3 (Fully Adjusted)** | SQ score (RCS) | (linear term) | 0.264 | 0.188 | −0.105 – 0.632 | 0.161 |
|  | SQ score (RCS) | (nonlinear term 1) | −1.050 | 0.614 | −2.255 – 0.153 | 0.087 |
|  | SQ score (RCS) | (nonlinear term 2) | 2.300 | 1.770 | −1.159 – 5.764 | 0.192 |
|  | Age (per year) | Age | −0.003 | 0.005 | −0.014 – 0.007 | 0.506 |
|  | Arthritis | Yes vs No | 0.296 | 0.082 | 0.135 – 0.457 | <0.001 |
|  | Asthma | Yes vs No | 0.107 | 0.197 | −0.278 – 0.493 | 0.585 |
|  | BMI category | Obesity (≥28.0) vs Normal | −0.178 | 0.130 | −0.433 – 0.076 | 0.170 |
|  | BMI category | Overweight (24.0–27.9) vs Normal | −0.021 | 0.089 | −0.195 – 0.153 | 0.814 |
|  | BMI category | Underweight (<18.5) vs Normal | 0.313 | 0.148 | 0.022 – 0.604 | 0.035 |
|  | Diabetes | Yes vs No | −0.384 | 0.190 | −0.757 – −0.011 | 0.044 |
|  | Dyslipidaemia | Yes vs No | 0.243 | 0.140 | −0.030 – 0.517 | 0.081 |
|  | Education | High school or above vs lower | −0.202 | 0.151 | −0.497 – 0.094 | 0.181 |
|  | Education | Illiterate vs lower | 0.068 | 0.111 | −0.150 – 0.286 | 0.540 |
|  | Education | Literate vs lower | 0.031 | 0.114 | −0.193 – 0.255 | 0.788 |
|  | Education | Middle school vs lower | −0.129 | 0.117 | −0.358 – 0.100 | 0.269 |
|  | Heart disease | Yes vs No | 0.387 | 0.124 | 0.145 – 0.629 | 0.002 |
|  | Hypertension | Yes vs No | −0.292 | 0.099 | −0.486 – −0.097 | 0.003 |
|  | Kidney disease | Yes vs No | 0.379 | 0.159 | 0.066 – 0.691 | 0.017 |
|  | Liver disease | Yes vs No | 0.483 | 0.199 | 0.093 – 0.872 | 0.015 |
|  | Lung disease | Yes vs No | 0.139 | 0.142 | −0.139 – 0.417 | 0.327 |
|  | Memory disorder | Yes vs No | −1.320 | 0.712 | −2.712 – 0.081 | 0.065 |
|  | Pain | Yes vs No | 0.184 | 0.085 | 0.017 – 0.351 | 0.031 |
|  | Psychological disorder | Yes vs No | 0.340 | 0.415 | −0.474 – 1.153 | 0.413 |
|  | Sex | Sex = Male (vs Female) | −0.096 | 0.119 | −0.328 – 0.137 | 0.420 |
|  | Stroke | Yes vs No | −0.060 | 0.286 | −0.621 – 0.500 | 0.833 |
|  | Drinking status | Ever vs Current | 0.436 | 0.138 | 0.165 – 0.707 | 0.002 |
|  | Drinking status | Never vs Current | 0.061 | 0.099 | −0.133 – 0.254 | 0.537 |
|  | Residence | Rural vs Urban | −0.032 | 0.085 | −0.198 – 0.134 | 0.708 |
|  | Marital status | Other vs Married | 0.251 | 0.126 | 0.004 – 0.498 | 0.046 |
|  | Smoking status | Ever vs Current | −0.146 | 0.157 | −0.453 – 0.161 | 0.350 |
|  | Smoking status | Never vs Current | −0.011 | 0.113 | −0.232 – 0.210 | 0.923 |

***Notes*: β values are Cox log-hazard coefficients; HR = exp(β). SQ score was modeled using restricted cubic splines (RCS) with one linear and two nonlinear terms. Higher SQ indicates better sleep. Individual spline coefficients should not be interpreted separately; the SQ association should be evaluated using overall and nonlinearity tests. Model 0 was unadjusted. Model 1 adjusted for age and sex. Model 2 further adjusted for education, residence, marital status, BMI, smoking status, and drinking status. Model 3 included all Model 2 covariates and additionally adjusted for chronic comorbidities. Reference groups were female sex, urban residence, married status, normal BMI, current smoking, current drinking, lower education, and no chronic comorbidity; age was modeled per 1-year increase. β, coefficient; SE, standard error; CI, confidence interval; HR, hazard ratio; RCS, restricted cubic spline; SQ, sleep quality; BMI, body mass index.**

# Supplementary Table 8. RCS–Cox models for the association between CESD-10 score and incident digestive disease risk

| **Model** | **Variable** | **Term** | **β (Estimate)** | **SE** | **95% CI (Lower, Upper)** | ***P* value** |
| --- | --- | --- | --- | --- | --- | --- |
| **Model 0 (Crude)** | CESD-10 score | (linear term) | 0.060 | 0.047 | −0.033 – 0.153 | 0.204 |
|  |  | (nonlinear term 1) | 0.036 | 0.245 | −0.444 – 0.516 | 0.883 |
|  |  | (nonlinear term 2) | −0.153 | 0.558 | −1.248 – 0.941 | 0.784 |
| **Model 1 (Age, Sex)** | CESD-10 score | (linear term) | 0.059 | 0.047 | −0.034 – 0.152 | 0.214 |
|  |  | (nonlinear term 1) | 0.023 | 0.245 | −0.458 – 0.503 | 0.926 |
|  |  | (nonlinear term 2) | −0.118 | 0.559 | −1.213 – 0.977 | 0.833 |
|  | Age | Age (per year) | 0.006 | 0.005 | −0.003 – 0.015 | 0.171 |
|  | Sex | Sex = Male | −0.190 | 0.076 | −0.339 – −0.042 | 0.012 |
| **Model 2 (+ socio-demographic)** | CESD-10 score | (linear term) | 0.060 | 0.048 | −0.033 – 0.154 | 0.204 |
|  |  | (nonlinear term 1) | −0.019 | 0.246 | −0.501 – 0.463 | 0.939 |
|  |  | (nonlinear term 2) | −0.016 | 0.560 | −1.114 – 1.082 | 0.977 |
|  | Age | Age (per year) | −0.001 | 0.005 | −0.011 – 0.009 | 0.810 |
|  | Sex | Sex = Male | −0.152 | 0.119 | −0.385 – 0.081 | 0.200 |
|  | Education level | High school or above | −0.174 | 0.150 | −0.468 – 0.120 | 0.246 |
|  |  | Illiterate | 0.025 | 0.110 | −0.191 – 0.241 | 0.820 |
|  |  | Literate | 0.042 | 0.114 | −0.181 – 0.265 | 0.712 |
|  |  | Middle school | −0.132 | 0.116 | −0.360 – 0.096 | 0.255 |
|  | Residence | Rural village | −0.064 | 0.084 | −0.228 – 0.101 | 0.446 |
|  | Marital status | Other marital status | 0.183 | 0.125 | −0.062 – 0.428 | 0.142 |
|  | BMI | Underweight (<18.5) | 0.363 | 0.148 | 0.073 – 0.653 | 0.014 |
|  |  | Overweight (24.0–27.9) | −0.015 | 0.088 | −0.187 – 0.158 | 0.866 |
|  |  | Obesity (≥28.0) | −0.158 | 0.126 | −0.404 – 0.088 | 0.209 |
|  | Smoke status | Ever smoker | −0.113 | 0.156 | −0.418 – 0.192 | 0.468 |
|  |  | Never smoker | −0.021 | 0.113 | −0.243 – 0.200 | 0.852 |
|  | Drinking status | Ever drinker | 0.394 | 0.137 | 0.126 – 0.662 | 0.004 |
|  |  | Never drinker | 0.053 | 0.100 | −0.142 – 0.248 | 0.594 |
| **Model 3 (Fully Adjusted)** | CESD-10 score | (linear term) | 0.063 | 0.048 | −0.030 – 0.156 | 0.184 |
|  |  | (nonlinear term 1) | −0.097 | 0.246 | −0.579 – 0.386 | 0.694 |
|  |  | (nonlinear term 2) | 0.171 | 0.562 | −0.930 – 1.272 | 0.761 |
|  | Age | Age (per year) | −0.003 | 0.005 | −0.013 – 0.007 | 0.593 |
|  | Sex | Sex = Male | −0.115 | 0.119 | −0.348 – 0.119 | 0.336 |
|  | Education level | High school or above | −0.188 | 0.151 | −0.484 – 0.107 | 0.212 |
|  |  | Illiterate | 0.052 | 0.111 | −0.166 – 0.270 | 0.638 |
|  |  | Literate | 0.012 | 0.114 | −0.212 – 0.236 | 0.917 |
|  |  | Middle school | −0.120 | 0.117 | −0.349 – 0.109 | 0.303 |
|  | Residence | Rural village | −0.086 | 0.085 | −0.252 – 0.080 | 0.309 |
|  | Marital status | Other marital status | 0.228 | 0.126 | −0.019 – 0.475 | 0.070 |
|  | BMI | Underweight (<18.5) | 0.316 | 0.149 | 0.025 – 0.607 | 0.033 |
|  |  | Overweight (24.0–27.9) | −0.016 | 0.089 | −0.191 – 0.159 | 0.859 |
|  |  | Obesity (≥28.0) | −0.154 | 0.130 | −0.409 – 0.102 | 0.238 |
|  | Smoke status | Ever smoker | −0.147 | 0.157 | −0.454 – 0.160 | 0.349 |
|  |  | Never smoker | −0.006 | 0.114 | −0.228 – 0.217 | 0.961 |
|  | Drinking status | Ever drinker | 0.402 | 0.138 | 0.131 – 0.673 | 0.004 |
|  |  | Never drinker | 0.061 | 0.099 | −0.133 – 0.254 | 0.538 |
|  | Hypertension | Yes | −0.274 | 0.099 | −0.469 – −0.080 | 0.006 |
|  | Diabetes | Yes | −0.400 | 0.190 | −0.772 – −0.027 | 0.035 |
|  | Dyslipidaemia | Yes | 0.236 | 0.139 | −0.037 – 0.510 | 0.090 |
|  | Heart disease | Yes | 0.370 | 0.123 | 0.128 – 0.611 | 0.003 |
|  | Liver disease | Yes | 0.497 | 0.199 | 0.107 – 0.888 | 0.013 |
|  | Lung disease | Yes | 0.136 | 0.141 | −0.141 – 0.414 | 0.335 |
|  | Stroke | Yes | −0.066 | 0.287 | −0.628 – 0.496 | 0.818 |
|  | Kidney disease | Yes | 0.371 | 0.160 | 0.058 – 0.683 | 0.020 |
|  | Asthma | Yes | 0.089 | 0.196 | −0.294 – 0.473 | 0.649 |
|  | Arthritis | Yes | 0.286 | 0.082 | 0.124 – 0.447 | <0.001 |
|  | Pain | Yes | 0.147 | 0.088 | −0.026 – 0.320 | 0.095 |
|  | Memory disease | Yes | −1.350 | 0.713 | −2.744 – 0.049 | 0.059 |
|  | Psychiatric disease | Yes | 0.214 | 0.415 | −0.599 – 1.028 | 0.606 |

*Note*: β values are Cox log-hazard coefficients; HR = exp(β). CESD-10 score was modeled using restricted cubic splines (RCS) with one linear and two nonlinear terms. Individual spline coefficients should not be interpreted separately; the CESD-10 association should be evaluated using overall and nonlinearity tests. Model 0 was unadjusted. Model 1 adjusted for age and sex. Model 2 further adjusted for education, residence, marital status, BMI, smoking status, and drinking status. Model 3 included all Model 2 covariates and additionally adjusted for chronic comorbidities. Reference groups were female sex, urban residence, married status, normal BMI, current smoking, current drinking, lower education, and no chronic comorbidity; age was modeled per 1-year increase. CESD-10, 10-item Center for Epidemiologic Studies Depression Scale; β, coefficient; SE, standard error; CI, confidence interval; HR, hazard ratio; RCS, restricted cubic spline; BMI, body mass index; SQ, sleep quality.

# Supplementary Table 9. Exploratory mediation analysis of the association between SQ and incident digestive disease with depressive symptoms as the mediator

| **Model** | **ACME** | **95% CI (ACME)** | ***P* (ACME)** | **ADE** | **95% CI (ADE)** | ***P* (ADE)** | **TE** | **95% CI (TE)** | ***P* (TE)** | **PM** | **95% CI (PM)** |
| --- | --- | --- | --- | --- | --- | --- | --- | --- | --- | --- | --- |
| Model 0 (Crude) | 0.0172 | 0.0074 – 0.0275 | <0.001 | 0.0764 | 0.0471 – 0.1050 | <0.001 | 0.0991 | 0.0726 – 0.1260 | <0.001 | 0.175 | 0.0709 – 0.308 |
| Model 1 | 0.0159 | 0.0073 – 0.0263 | <0.001 | 0.0732 | 0.0445 – 0.1020 | <0.001 | 0.0942 | 0.0679 – 0.1200 | <0.001 | 0.171 | 0.0715 – 0.314 |
| Model 2 | 0.0124 | 0.0030 – 0.0218 | 0.004 | 0.0741 | 0.0460 – 0.1050 | <0.001 | 0.0902 | 0.0627 – 0.1180 | <0.001 | 0.137 | 0.0316 – 0.274 |
| Model 3 (Fully) | 0.0063 | −0.0016 – 0.0154 | 0.106 | 0.0702 | 0.0432 – 0.0979 | <0.001 | 0.0782 | 0.0530 – 0.1050 | <0.001 | 0.078 | −0.0224 – 0.213 |

***Notes*:** ACME represents the model-based indirect effect of SQ on incident digestive disease through depressive symptoms. ADE represents the direct effect not operating through depressive symptoms, while TE represents the total effect. PM indicates the proportion of the total effect accounted for by the indirect association through depressive symptoms. Depressive symptoms were defined as CESD-10 score ≥10. Model 0 was unadjusted. Model 1 adjusted for age and sex. Model 2 further adjusted for education level, residence, marital status, BMI, smoking status, and drinking status. Model 3 additionally adjusted for hypertension, diabetes, dyslipidaemia, heart disease, liver disease, lung disease, stroke, kidney disease, asthma, arthritis, pain, memory disorder, and psychiatric disorder. These mediation analyses were exploratory and assumption-dependent and were not interpreted as confirmation of causal pathways. ACME, average causal mediation effect; ADE, average direct effect; TE, total effect; PM, proportion mediated; SQ, sleep quality; CESD-10, 10-item Center for Epidemiologic Studies Depression Scale; BMI, body mass index; CI, confidence interval.

# Supplementary Table 10. Exploratory mediation analysis of the association between depressive symptoms and incident digestive disease with SQ as the mediator

| **Model** | **ACME** | **95% CI (ACME)** | ***P* (ACME)** | **ADE** | **95% CI (ADE)** | ***P* (ADE)** | **TE** | **95% CI (TE)** | ***P* (TE)** | **PM** | **95% CI (PM)** |
| --- | --- | --- | --- | --- | --- | --- | --- | --- | --- | --- | --- |
| Model 0 (Crude) | 0.0294 | 0.0178 – 0.0413 | <0.001 | 0.0571 | 0.0283 – 0.0901 | <0.001 | 0.0932 | 0.0640 – 0.1250 | <0.001 | 0.314 | 0.173 – 0.538 |
| Model 1 | 0.0278 | 0.0165 – 0.0397 | <0.001 | 0.0534 | 0.0240 – 0.0839 | 0.001 | 0.0873 | 0.0579 – 0.1180 | <0.001 | 0.320 | 0.179 – 0.551 |
| Model 2 | 0.0281 | 0.0173 – 0.0409 | <0.001 | 0.0420 | 0.0100 – 0.0730 | 0.005 | 0.0752 | 0.0434 – 0.1060 | <0.001 | 0.374 | 0.206 – 0.759 |
| Model 3 (Fully) | 0.0248 | 0.0147 – 0.0362 | <0.001 | 0.0251 | −0.0042 – 0.0565 | 0.111 | 0.0522 | 0.0218 – 0.0831 | <0.001 | 0.473 | 0.243 – 1.210 |

***Notes*:** Depressive symptoms were defined as CESD-10 score ≥10. ACME represents the model-based indirect effect of depressive symptoms on incident digestive disease through SQ. ADE represents the direct effect not operating through SQ, while TE represents the total effect. PM indicates the proportion of the total effect accounted for by the indirect association through SQ. Model 0 was unadjusted; Model 1 adjusted for age and sex; Model 2 further adjusted for education level, residence, marital status, BMI, smoking status, and drinking status; Model 3 additionally adjusted for hypertension, diabetes, dyslipidaemia, heart disease, liver disease, lung disease, stroke, kidney disease, asthma, arthritis, pain, memory disorder, and psychiatric disorder. These mediation analyses were exploratory and assumption-dependent and were not interpreted as confirmation of causal pathways. ACME, average causal mediation effect; ADE, average direct effect; TE, total effect; PM, proportion mediated; SQ, sleep quality; CESD-10, 10-item Center for Epidemiologic Studies Depression Scale; BMI, body mass index; CI, confidence interval.

# Supplementary Table 11. Associations of joint trajectory groups and covariates with incident digestive disease risk across Cox proportional hazards models

| **Variable** | **Level / Comparison** | **Model 0 (Crude)** | **Model 1 (Age, Sex)** | **Model 2 (+ socio-demographic)** | **Model 3 (Fully adjusted)** |
| --- | --- | --- | --- | --- | --- |
| **Age** | Continuous | – | 1.01 (1.00–1.02), *P*=0.155 | 1.00 (0.99–1.01), *P*=0.726 | 1.00 (0.99–1.01), *P*=0.519 |
| **Arthritis** | Yes vs No | – | – | – | 1.32 (1.12–1.55), *P*<0.001 |
| **Asthma** | Yes vs No | – | – | – | 1.08 (0.73–1.58), *P*=0.708 |
| **BMI category** | Obesity (≥28.0) vs Normal | – | – | 0.85 (0.67–1.09), *P*=0.200 | 0.86 (0.66–1.11), *P*=0.237 |
|  | Overweight (24.0–27.9) vs Normal | – | – | 0.98 (0.82–1.16), *P*=0.808 | 0.97 (0.82–1.16), *P*=0.767 |
|  | Underweight (<18.5) vs Normal | – | – | 1.47 (1.10–1.96), *P*=0.009 | 1.39 (1.04–1.86), *P*=0.025 |
| **Diabetes** | Yes vs No | – | – | – | 0.66 (0.46–0.96), *P*=0.030 |
| **Dyslipidaemia** | Yes vs No | – | – | – | 1.27 (0.97–1.67), *P*=0.086 |
| **Education level** | High school or above vs Elementary | – | – | 0.81 (0.61–1.09), *P*=0.166 | 0.82 (0.61–1.10), *P*=0.177 |
|  | Illiterate vs Elementary | – | – | 1.03 (0.83–1.28), *P*=0.785 | 1.05 (0.84–1.31), *P*=0.658 |
|  | Literate vs Elementary | – | – | 1.04 (0.83–1.30), *P*=0.723 | 1.00 (0.80–1.26), *P*=0.972 |
|  | Middle school vs Elementary | – | – | 0.86 (0.69–1.08), *P*=0.206 | 0.88 (0.70–1.10), *P*=0.265 |
| **Heart disease** | Yes vs No | – | – | – | 1.47 (1.16–1.87), *P*=0.002 |
| **Hypertension** | Yes vs No | – | – | – | 0.76 (0.63–0.92), *P*=0.006 |
| **Kidney disease** | Yes vs No | – | – | – | 1.48 (1.08–2.02), *P*=0.015 |
| **Liver disease** | Yes vs No | – | – | – | 1.61 (1.09–2.38), *P*=0.017 |
| **Lung disease** | Yes vs No | – | – | – | 1.13 (0.86–1.50), *P*=0.376 |
| **Memory disease** | Yes vs No | – | – | – | 0.26 (0.06–1.06), *P*=0.060 |
| **Pain** | Yes vs No | – | – | – | 1.23 (1.04–1.45), *P*=0.017 |
| **Psychiatric disease** | Yes vs No | – | – | – | 1.27 (0.56–2.87), *P*=0.565 |
| **Sex** | Male vs Female | – | 0.84 (0.73–0.98), *P*=0.027 | 0.87 (0.69–1.10), *P*=0.239 | 0.90 (0.71–1.14), *P*=0.378 |
| **Stroke** | Yes vs No | – | – | – | 0.96 (0.55–1.68), *P*=0.884 |
| **Drinking status** | Ever vs Current | – | – | 1.48 (1.13–1.93), *P*=0.004 | 1.48 (1.13–1.94), *P*=0.005 |
|  | Never vs Current | – | – | 1.04 (0.85–1.26), *P*=0.705 | 1.05 (0.87–1.28), *P*=0.615 |
| **Residence** | Rural vs Urban | – | – | 0.96 (0.82–1.14), *P*=0.670 | 0.94 (0.80–1.11), *P*=0.491 |
| **Marital status** | Other vs Married | – | – | 1.24 (0.97–1.58), *P*=0.089 | 1.28 (1.00–1.64), *P*=0.047 |
| **Smoking status** | Ever vs Current | – | – | 0.90 (0.67–1.23), *P*=0.512 | 0.87 (0.64–1.18), *P*=0.375 |
|  | Never vs Current | – | – | 0.97 (0.78–1.21), *P*=0.810 | 0.99 (0.79–1.23), *P*=0.908 |
| **Joint trajectory group** | Group 2 (moderate worsening) vs Group 1 | 1.66 (1.37–2.00), *P*<0.001 | 1.61 (1.33–1.94), *P*<0.001 | 1.56 (1.29–1.88), *P*<0.001 | 1.50 (1.24–1.82), *P*<0.001 |
|  | Group 3 (marked worsening; lower SQ) vs Group 1 | 1.84 (1.55–2.19), *P*<0.001 | 1.78 (1.49–2.11), *P*<0.001 | 1.68 (1.41–2.00), *P*<0.001 | 1.48 (1.23–1.78), *P*<0.001 |

*Notes*: Model 0 was unadjusted. Model 1 adjusted for age and sex. Model 2 further adjusted for education level, residence, marital status, BMI category, smoking status, and drinking status. Model 3 included all Model 2 covariates and additionally adjusted for chronic comorbidities. Reference categories were female sex, elementary education, urban residence, married status, normal BMI, current smoking, and current drinking. For binary comorbidities, “No” served as the reference category. Age was modeled per 1-year increase. “—” indicates that the covariate was not included in that model. Joint trajectory Group 1, characterized by stable/low depressive symptoms and higher SQ, served as the reference group; Group 2 represented moderate worsening; and Group 3 represented marked worsening with lower SQ. HR, hazard ratio; CI, confidence interval; BMI, body mass index; SQ, sleep quality; Ref, reference.

# Supplementary Table 12. Baseline characteristics stratified by SQ group

| **Variable** | **Overall (n=3711)** | **Good (score=4, n=2227)** | **Moderate/Poor (score≤3, n=1484)** | ***P* value** |
| --- | --- | --- | --- | --- |
| **Age, mean (SD)** | 57.71 (8.23) | 57.53 (8.29) | 57.99 (8.13) | 0.096 |
| **CESD-10 score, mean (SD)** | 6.94 (5.66) | 4.85 (4.41) | 10.07 (5.88) | <0.001 |
| **SQ score** | 3.25 (1.06) | 4.00 (0.00) | 2.12 (0.83) | <0.001 |
| **Sex, n (%)** |  |  |  | <0.001 |
| Female | 1824 (49.2) | 988 (44.4) | 836 (56.3) |  |
| Male | 1887 (50.8) | 1239 (55.6) | 648 (43.7) |  |
| **Education level, n (%)** |  |  |  | <0.001 |
| Illiterate | 888 (23.9) | 477 (21.4) | 411 (27.7) |  |
| Literate | 683 (18.4) | 406 (18.2) | 277 (18.7) |  |
| Elementary school | 876 (23.6) | 533 (23.9) | 343 (23.1) |  |
| Middle school | 828 (22.3) | 511 (22.9) | 317 (21.4) |  |
| High school or above | 436 (11.7) | 300 (13.5) | 136 (9.2) |  |
| **Residence, n (%)** |  |  |  | 0.282 |
| Urban community | 1266 (34.1) | 744 (33.4) | 522 (35.2) |  |
| Rural village | 2445 (65.9) | 1483 (66.6) | 962 (64.8) |  |
| **Marital status, n (%)** |  |  |  | 0.011 |
| Married | 3388 (91.3) | 2055 (92.3) | 1333 (89.8) |  |
| Other | 323 (8.7) | 172 (7.7) | 151 (10.2) |  |
| **BMI, n (%)** |  |  |  | <0.001 |
| Underweight (<18.5) | 182 (4.9) | 87 (3.9) | 95 (6.4) |  |
| Normal (18.5–23.9) | 1940 (52.3) | 1164 (52.3) | 776 (52.3) |  |
| Overweight (24.0–27.9) | 1118 (30.1) | 715 (32.1) | 403 (27.2) |  |
| Obesity (≥28.0) | 471 (12.7) | 261 (11.7) | 210 (14.2) |  |
| **Smoking status, n (%)** |  |  |  | <0.001 |
| Never | 2193 (59.1) | 1250 (56.1) | 943 (63.5) |  |
| Ever | 311 (8.4) | 202 (9.1) | 109 (7.3) |  |
| Current | 1207 (32.5) | 775 (34.8) | 432 (29.1) |  |
| **Drinking status, n (%)** |  |  |  | <0.001 |
| Never | 2202 (59.3) | 1267 (56.9) | 935 (63.0) |  |
| Ever | 293 (7.9) | 176 (7.9) | 117 (7.9) |  |
| Current | 1216 (32.8) | 784 (35.2) | 432 (29.1) |  |
| **Depressive symptoms, n (%)** |  |  |  | <0.001 |
| Yes | 1018 (27.4) | 315 (14.1) | 703 (47.4) |  |
| **Hypertension, n (%)** |  |  |  | <0.001 |
| Yes | 869 (23.4) | 466 (20.9) | 403 (27.2) |  |
| **Diabetes, n (%)** |  |  |  | 0.041 |
| Yes | 204 (5.5) | 108 (4.8) | 96 (6.5) |  |
| **Dyslipidaemia, n (%)** |  |  |  | 0.052 |
| Yes | 290 (7.8) | 158 (7.1) | 132 (8.9) |  |
| **Heart disease, n (%)** |  |  |  | 0.004 |
| Yes | 314 (8.5) | 164 (7.4) | 150 (10.1) |  |
| **Liver disease, n (%)** |  |  |  | 0.165 |
| Yes | 88 (2.4) | 46 (2.1) | 42 (2.8) |  |
| **Lung disease, n (%)** |  |  |  | 0.001 |
| Yes | 291 (7.8) | 148 (6.6) | 143 (9.6) |  |
| **Stroke, n (%)** |  |  |  | 0.007 |
| Yes | 67 (1.8) | 29 (1.3) | 38 (2.6) |  |
| **Kidney disease, n (%)** |  |  |  | 0.062 |
| Yes | 149 (4.0) | 78 (3.5) | 71 (4.8) |  |
| **Asthma, n (%)** |  |  |  | 0.170 |
| Yes | 144 (3.9) | 78 (3.5) | 66 (4.4) |  |
| **Arthritis, n (%)** |  |  |  | <0.001 |
| Yes | 969 (26.1) | 501 (22.5) | 468 (31.5) |  |
| **Pain, n (%)** |  |  |  | <0.001 |
| Yes | 944 (25.4) | 427 (19.2) | 517 (34.8) |  |
| **Memory disease, n (%)** |  |  |  | 0.614 |
| Yes | 28 (0.8) | 15 (0.7) | 13 (0.9) |  |
| **Psychiatric disease, n (%)** |  |  |  | 0.106 |
| Yes | 22 (0.6) | 9 (0.4) | 13 (0.9) |  |
| **Incident digestive disease, n (%)** | 722 (19.5) | 345 (15.5) | 377 (25.4) | <0.001 |

*Notes*: Values are presented as mean (SD) or n (%). P values compare Good SQ (SQ = 4) with Moderate/Poor SQ (SQ ≤3) using t tests or Wilcoxon rank-sum tests for continuous variables, and χ² or Fisher exact tests for categorical variables, as appropriate. SQ score is a composite index ranging from 0 to 4, with higher scores indicating better SQ. CESD-10 score ranges from 0 to 30, and depressive symptoms were defined as CESD-10 score ≥10. BMI categories followed Chinese adult cut points: <18.5 kg/m² for underweight, 18.5–23.9 kg/m² for normal weight, 24.0–27.9 kg/m² for overweight, and ≥28.0 kg/m² for obesity. Incident digestive disease indicates newly reported digestive disease during follow-up. SQ, sleep quality; CESD-10, 10-item Center for Epidemiologic Studies Depression Scale; SD, standard deviation; BMI, body mass index.

# Supplementary Table 13. Baseline characteristics stratified by depressive symptom severity

| **Variable** | **Overall (n=3711)** | **CESD-10 < 12 (n = 3,002)** | **CESD-10 ≥ 12 (n = 709)** | ***P* value** |
| --- | --- | --- | --- | --- |
| **Age, mean (SD)** | 57.71 (8.23) | 57.51 (8.23) | 58.54 (8.18) | 0.003 |
| **CESD-10 score, mean (SD)** | 6.94 (5.66) | 4.75 (3.28) | 16.22 (3.93) | <0.001 |
| **SQ score, mean (SD)** | 3.25 (1.06) | 3.44 (0.93) | 2.44 (1.17) | <0.001 |
| **Sex, n (%)** |  |  |  | <0.001 |
| Female | 1,824 (49.2) | 1,404 (46.8) | 420 (59.2) |  |
| Male | 1,887 (50.8) | 1,598 (53.2) | 289 (40.8) |  |
| **Education level, n (%)** |  |  |  | <0.001 |
| Illiterate | 888 (23.9) | 661 (22.0) | 227 (32.0) |  |
| Literate | 683 (18.4) | 522 (17.4) | 161 (22.7) |  |
| Elementary school | 876 (23.6) | 715 (23.8) | 161 (22.7) |  |
| Middle school | 828 (22.3) | 700 (23.3) | 128 (18.1) |  |
| High school or above | 436 (11.7) | 404 (13.5) | 32 (4.5) |  |
| **Residence** |  |  |  | <0.001 |
| Urban community | 1,266 (34.1) | 1,096 (36.5) | 170 (24.0) |  |
| Rural village | 2,445 (65.9) | 1,906 (63.5) | 539 (76.0) |  |
| **Marital status** |  |  |  | <0.001 |
| Married | 3,388 (91.3) | 2,767 (92.2) | 621 (87.6) |  |
| Other | 323 (8.7) | 235 (7.8) | 88 (12.4) |  |
| **BMI category** |  |  |  | <0.001 |
| Underweight (<18.5) | 182 (4.9) | 120 (4.0) | 62 (8.7) |  |
| Normal (18.5–23.9) | 1,940 (52.3) | 1,563 (52.1) | 377 (53.2) |  |
| Overweight (24.0–27.9) | 1,118 (30.1) | 934 (31.1) | 184 (26.0) |  |
| Obesity (≥28.0) | 471 (12.7) | 385 (12.8) | 86 (12.1) |  |
| **Smoking status** |  |  |  | 0.115 |
| Never | 2,193 (59.1) | 1,750 (58.3) | 443 (62.5) |  |
| Ever | 311 (8.4) | 259 (8.6) | 52 (7.3) |  |
| Current | 1,207 (32.5) | 993 (33.1) | 214 (30.2) |  |
| **Drinking status** |  |  |  | <0.001 |
| Never | 2,202 (59.3) | 1,747 (58.2) | 455 (64.2) |  |
| Ever | 293 (7.9) | 219 (7.3) | 74 (10.4) |  |
| Current | 1,216 (32.8) | 1,036 (34.5) | 180 (25.4) |  |
| **SQ category** |  |  |  | <0.001 |
| Good | 2,227 (60.0) | 2,033 (67.7) | 194 (27.4) |  |
| Moderate | 611 (16.5) | 484 (16.1) | 127 (17.9) |  |
| Poor | 873 (23.5) | 485 (16.2) | 388 (54.7) |  |
| **Comorbidities, n (%)** |  |  |  | — |
| Hypertension | 869 (23.4) | 687 (22.9) | 182 (25.7) | 0.127 |
| Diabetes | 204 (5.5) | 163 (5.4) | 41 (5.8) | 0.780 |
| Dyslipidaemia | 290 (7.8) | 234 (7.8) | 56 (7.9) | 0.988 |
| Heart disease | 314 (8.5) | 236 (7.9) | 78 (11.0) | 0.009 |
| Lung disease | 291 (7.8) | 204 (6.8) | 87 (12.3) | <0.001 |
| Stroke | 67 (1.8) | 43 (1.4) | 24 (3.4) | 0.001 |
| Arthritis | 969 (26.1) | 685 (22.8) | 284 (40.1) | <0.001 |
| Pain | 944 (25.4) | 576 (19.2) | 368 (51.9) | <0.001 |
| **Incident digestive disease, n (%)** | 722 (19.5) | 521 (17.4) | 201 (28.3) | <0.001 |

*Notes*: Values are presented as mean (SD) or n (%). P values compare participants with CESD-10 <12 and CESD-10 ≥12 using t tests or Wilcoxon rank-sum tests for continuous variables, and χ² or Fisher exact tests for categorical variables, as appropriate. CESD-10 score ranges from 0 to 30; CESD-10 ≥12 was used to define higher depressive symptom severity in this sensitivity analysis. SQ score is a composite index ranging from 0 to 4, with higher scores indicating better SQ. BMI categories followed Chinese adult cut points: <18.5 kg/m² for underweight, 18.5–23.9 kg/m² for normal weight, 24.0–27.9 kg/m² for overweight, and ≥28.0 kg/m² for obesity. Incident digestive disease indicates newly reported digestive disease during follow-up. SQ, sleep quality; CESD-10, 10-item Center for Epidemiologic Studies Depression Scale; SD, standard deviation; BMI, body mass index.

# Supplementary Table 14. Baseline characteristics of participants without baseline comorbidities stratified by SQ category

| **Variable** | **Overall (n = 1,372)** | **Good (n = 955)** | **Moderate (n = 184)** | **Poor (n = 233)** | ***P* value** |
| --- | --- | --- | --- | --- | --- |
| **Age, mean (SD)** | 56.24 (8.20) | 56.10 (8.10) | 56.54 (8.62) | 56.59 (8.29) | 0.621 |
| **CESD-10 score** | 5.29 (4.60) | 3.99 (3.70) | 6.58 (4.18) | 9.56 (5.34) | <0.001 |
| **SQ score** | 3.45 (0.95) | 4.00 (0.00) | 3.00 (0.00) | 1.54 (0.50) | <0.001 |
| **Sex, n (%)** |  |  |  |  | 0.023 |
| Female | 634 (46.2) | 418 (43.8) | 95 (51.6) | 121 (51.9) |  |
| Male | 738 (53.8) | 537 (56.2) | 89 (48.4) | 112 (48.1) |  |
| **Education level, n (%)** |  |  |  |  | 0.564 |
| Illiterate | 316 (23.0) | 210 (22.0) | 48 (26.1) | 58 (24.9) |  |
| Literate | 215 (15.7) | 153 (16.0) | 23 (12.5) | 39 (16.7) |  |
| Elementary school | 327 (23.8) | 223 (23.4) | 47 (25.5) | 57 (24.5) |  |
| Middle school | 333 (24.3) | 233 (24.4) | 43 (23.4) | 57 (24.5) |  |
| High school or above | 181 (13.2) | 136 (14.2) | 23 (12.5) | 22 (9.4) |  |
| **Residence** |  |  |  |  | 0.034 |
| Urban community | 471 (34.3) | 309 (32.4) | 77 (41.8) | 85 (36.5) |  |
| Rural village | 901 (65.7) | 646 (67.6) | 107 (58.2) | 148 (63.5) |  |
| **Marital status** |  |  |  |  | 0.031 |
| Married | 1,255 (91.5) | 886 (92.8) | 162 (88.0) | 207 (88.8) |  |
| Other | 117 (8.5) | 69 (7.2) | 22 (12.0) | 26 (11.2) |  |
| **BMI category** |  |  |  |  | 0.152 |
| Underweight (<18.5) | 61 (4.4) | 38 (4.0) | 11 (6.0) | 12 (5.2) |  |
| Normal (18.5–23.9) | 828 (60.3) | 565 (59.2) | 111 (60.3) | 152 (65.2) |  |
| Overweight (24.0–27.9) | 376 (27.4) | 276 (28.9) | 52 (28.3) | 48 (20.6) |  |
| Obesity (≥28.0) | 107 (7.8) | 76 (8.0) | 10 (5.4) | 21 (9.0) |  |
| **Smoking status** |  |  |  |  | 0.085 |
| Never | 805 (58.7) | 542 (56.8) | 122 (66.3) | 141 (60.5) |  |
| Ever | 89 (6.5) | 65 (6.8) | 13 (7.1) | 11 (4.7) |  |
| Current | 478 (34.8) | 348 (36.4) | 49 (26.6) | 81 (34.8) |  |
| **Drinking status** |  |  |  |  | 0.003 |
| Never | 813 (59.3) | 543 (56.9) | 115 (62.5) | 155 (66.5) |  |
| Ever | 70 (5.1) | 45 (4.7) | 7 (3.8) | 18 (7.7) |  |
| Current | 489 (35.6) | 367 (38.4) | 62 (33.7) | 60 (25.8) |  |
| **Depressive symptoms** | 226 (16.5) | 78 (8.2) | 39 (21.2) | 109 (46.8) | <0.001 |
| **Incident digestive disease** | 220 (16.0) | 131 (13.7) | 39 (21.2) | 50 (21.5) | 0.002 |

*Notes*: Values are presented as mean (SD) or n (%). P values were calculated using ANOVA or Kruskal–Wallis tests for continuous variables, and χ² or Fisher exact tests for categorical variables, as appropriate, comparing the three SQ groups. The analytic subsample included participants without baseline comorbidities. SQ score is a composite index ranging from 0 to 4, with higher scores indicating better SQ. CESD-10 score ranges from 0 to 30, and depressive symptoms were defined as CESD-10 score ≥10. BMI categories followed Chinese adult cut points: <18.5 kg/m² for underweight, 18.5–23.9 kg/m² for normal weight, 24.0–27.9 kg/m² for overweight, and ≥28.0 kg/m² for obesity. Incident digestive disease indicates newly reported digestive disease during follow-up. SQ, sleep quality; CESD-10, 10-item Center for Epidemiologic Studies Depression Scale; SD, standard deviation; BMI, body mass index.

# Supplementary Table 15. SQ score by high modified CESD-9 burden status at baseline

| **High modified CESD-9 burden (modified CESD-9 ≥9)** | **n** | **Mean SQ** | **SD SQ** | **Median SQ** | **Q1** | **Q3** |
| --- | --- | --- | --- | --- | --- | --- |
| Low | 2,688 | 3.43 | 0.95 | 4 | 3 | 4 |
| High | 1,023 | 2.78 | 1.17 | 3 | 2 | 4 |

*Notes*: SQ, sleep quality; CESD-9, modified 9-item Center for Epidemiologic Studies Depression Scale; SD, standard deviation; Q1/Q3, first/third quartile. Modified CESD-9 was calculated by subtracting the restless-sleep item from the CESD-10 total score. High modified CESD-9 burden was defined as modified CESD-9 score ≥9 for sensitivity analyses only and was not interpreted as a clinical diagnosis of depression.

# Supplementary Table 16. Modified CESD-9 score by SQ category at baseline

| **SQ category** | **n** | **Mean modified CESD-9** | **SD modified CESD-9** | **Median modified CESD-9** | **Q1** | **Q3** | **High modified CESD-9 burden (modified CESD-9 ≥9), %** |
| --- | --- | --- | --- | --- | --- | --- | --- |
| Good | 2,227 | 4.85 | 4.41 | 4 | 1 | 7 | 18.1% |
| Moderate | 611 | 6.90 | 4.83 | 6 | 3 | 10 | 31.6% |
| Poor | 873 | 9.10 | 6.03 | 8 | 5 | 13 | 48.8% |

**Notes**: SQ, sleep quality; CESD-9, modified 9-item Center for Epidemiologic Studies Depression Scale; SD, standard deviation; Q1/Q3, first/third quartile. Modified CESD-9 was calculated by subtracting the restless-sleep item from the CESD-10 total score. High modified CESD-9 burden was defined as modified CESD-9 score ≥9 for sensitivity analyses only and was not interpreted as a clinical diagnosis of depression.

# Supplementary Table 17. Association between modified CESD-9 score and SQ across multivariable linear regression models

| **Model** | **Variable** | **Term** | **β (Estimate)** | **SE** | **95% CI (Lower, Upper)** | ***P* value** |
| --- | --- | --- | --- | --- | --- | --- |
| **Crude (unadjusted)** | Intercept | Intercept | 3.620 | 0.042 | 3.536 – 3.699 | <0.001 |
|  | Modified CESD-9 score | linear term | -0.033 | 0.022 | -0.076 – 0.010 | 0.132 |
|  | Modified CESD-9 score | nonlinear term 1 | -0.216 | 0.143 | -0.496 – 0.063 | 0.129 |
|  | Modified CESD-9 score | nonlinear term 2 | 0.413 | 0.281 | -0.138 – 0.964 | 0.142 |
| **Model 1 (Age, Sex)** | Intercept | Intercept | 3.660 | 0.121 | 3.426 – 3.901 | <0.001 |
|  | Modified CESD-9 score | linear term | -0.032 | 0.022 | -0.075 – 0.011 | 0.142 |
|  | Modified CESD-9 score | nonlinear term 1 | -0.208 | 0.142 | -0.487 – 0.071 | 0.144 |
|  | Modified CESD-9 score | nonlinear term 2 | 0.395 | 0.280 | -0.155 – 0.945 | 0.159 |
|  | Age | Age | -0.002 | 0.002 | -0.006 – 0.002 | 0.287 |
|  | Sex | Male vs Female | 0.132 | 0.033 | 0.067 – 0.197 | <0.001 |
| **Model 2 (+ socio-demographic)** | Intercept | Intercept | 3.540 | 0.136 | 3.272 – 3.806 | <0.001 |
|  | Modified CESD-9 score | linear term | -0.032 | 0.022 | -0.074 – 0.011 | 0.146 |
|  | Modified CESD-9 score | nonlinear term 1 | -0.217 | 0.142 | -0.496 – 0.062 | 0.127 |
|  | Modified CESD-9 score | nonlinear term 2 | 0.414 | 0.281 | -0.136 – 0.964 | 0.140 |
|  | Age | Age | -0.001 | 0.002 | -0.005 – 0.003 | 0.588 |
|  | Sex | Male vs Female | 0.106 | 0.036 | 0.035 – 0.176 | 0.003 |
|  | Education level | High school or above | 0.110 | 0.060 | -0.008 – 0.227 | 0.067 |
|  | Education level | Illiterate | -0.020 | 0.050 | -0.118 – 0.078 | 0.690 |
|  | Education level | Literate | 0.042 | 0.051 | -0.058 – 0.142 | 0.413 |
|  | Marital status | Other vs Married | -0.024 | 0.060 | -0.142 – 0.095 | 0.696 |
|  | Residence | Rural vs Urban | 0.105 | 0.036 | 0.034 – 0.176 | 0.004 |
| **Model 3 (+ lifestyle & comorbidities)** | Intercept | Intercept | 3.450 | 0.149 | 3.155 – 3.737 | <0.001 |
|  | Modified CESD-9 score | linear term | -0.026 | 0.022 | -0.069 – 0.016 | 0.227 |
|  | Modified CESD-9 score | nonlinear term 1 | -0.194 | 0.142 | -0.472 – 0.085 | 0.172 |
|  | Modified CESD-9 score | nonlinear term 2 | 0.366 | 0.280 | -0.183 – 0.915 | 0.191 |
|  | Age | Age | 0.001 | 0.002 | -0.004 – 0.005 | 0.811 |
|  | Sex | Male vs Female | 0.102 | 0.051 | 0.001 – 0.202 | 0.047 |
|  | Education level | High school or above | 0.090 | 0.060 | -0.027 – 0.208 | 0.132 |
|  | Education level | Illiterate | -0.016 | 0.050 | -0.114 – 0.082 | 0.750 |
|  | Education level | Literate | 0.054 | 0.051 | -0.045 – 0.154 | 0.286 |
|  | Education level | Middle school | 0.007 | 0.049 | -0.090 – 0.103 | 0.893 |
|  | Marital status | Other vs Married | -0.038 | 0.060 | -0.156 – 0.081 | 0.531 |
|  | Residence | Rural vs Urban | 0.112 | 0.036 | 0.041 – 0.184 | 0.002 |
|  | BMI category | Obesity vs Normal | -0.042 | 0.054 | -0.148 – 0.064 | 0.437 |
|  | BMI category | Overweight vs Normal | 0.116 | 0.039 | 0.040 – 0.192 | 0.003 |
|  | BMI category | Underweight vs Normal | -0.149 | 0.078 | -0.301 – 0.003 | 0.055 |
|  | Smoking status | Ever vs Current | 0.078 | 0.064 | -0.048 – 0.203 | 0.224 |
|  | Smoking status | Never vs Current | 0.033 | 0.048 | -0.062 – 0.127 | 0.500 |
|  | Drinking status | Ever vs Current | 0.054 | 0.066 | -0.075 – 0.184 | 0.410 |
|  | Drinking status | Never vs Current | -0.012 | 0.042 | -0.095 – 0.070 | 0.770 |
|  | Hypertension | Yes vs No | -0.083 | 0.042 | -0.164 – -0.002 | 0.046 |
|  | Diabetes | Yes vs No | -0.080 | 0.074 | -0.225 – 0.065 | 0.281 |
|  | Dyslipidaemia | Yes vs No | 0.082 | 0.065 | -0.045 – 0.209 | 0.207 |
|  | Heart disease | Yes vs No | -0.068 | 0.061 | -0.188 – 0.052 | 0.269 |
|  | Liver disease | Yes vs No | -0.181 | 0.107 | -0.391 – 0.030 | 0.092 |
|  | Lung disease | Yes vs No | -0.117 | 0.069 | -0.252 – 0.018 | 0.088 |
|  | Stroke | Yes vs No | -0.070 | 0.125 | -0.315 – 0.176 | 0.579 |
|  | Kidney disease | Yes vs No | -0.032 | 0.084 | -0.197 – 0.132 | 0.699 |
|  | Asthma | Yes vs No | 0.074 | 0.095 | -0.112 – 0.261 | 0.435 |
|  | Arthritis | Yes vs No | -0.055 | 0.039 | -0.130 – 0.021 | 0.154 |
|  | Pain | Yes vs No | -0.195 | 0.041 | -0.275 – -0.114 | <0.001 |
|  | Memory disease | Yes vs No | 0.036 | 0.191 | -0.339 – 0.411 | 0.852 |
|  | Psychiatric disease | Yes vs No | 0.118 | 0.214 | -0.301 – 0.537 | 0.581 |

*Notes*: Results are from multivariable linear regression models with SQ score as the dependent variable. Modified CESD-9 score was modeled using restricted cubic splines with four knots. Modified CESD-9 was calculated by subtracting the restless-sleep item from the CESD-10 total score. Individual spline coefficients should not be interpreted separately; the overall and nonlinear associations should be evaluated using joint tests. Model 0 was unadjusted. Model 1 adjusted for age and sex. Model 2 further adjusted for marital status, education level, and residence. Model 3 additionally adjusted for BMI, smoking status, drinking status, and chronic comorbidities. Because higher SQ indicates better sleep, a negative β indicates an association with lower SQ, that is, poorer SQ. SE, standard error; CI, confidence interval; SQ, sleep quality; CESD-9, modified 9-item Center for Epidemiologic Studies Depression Scale; CESD-10, 10-item Center for Epidemiologic Studies Depression Scale; BMI, body mass index.

# **Supplementary Table 18. Exploratory bidirectional mediation analyses using modified CESD-9 after excluding the restless-sleep item**

|  | **Model** | **ACME (95% CI), P** | **ADE (95% CI), P** | **Total effect (95% CI), P** | **PM (95% CI), P** |
| --- | --- | --- | --- | --- | --- |
| Poorer SQ → high modified CESD-9 burden → incident digestive disease | Model 0 | 0.0115 (0.0052–0.0188), P < 0.001 | 0.0834 (0.0561–0.1121), P < 0.001 | 0.0990 (0.0725–0.1266), P < 0.001 | 0.115 (0.049–0.209), P < 0.001 |
| Poorer SQ → high modified CESD-9 burden → incident digestive disease | Model 1 | 0.0104 (0.0042–0.0174), P < 0.001 | 0.0805 (0.0532–0.1085), P < 0.001 | 0.0944 (0.0676–0.1214), P < 0.001 | 0.109 (0.042–0.206), P < 0.001 |
| Poorer SQ → high modified CESD-9 burden → incident digestive disease | Model 2 | 0.0081 (0.0021–0.0146), P = 0.009 | 0.0804 (0.0532–0.1083), P < 0.001 | 0.0911 (0.0644–0.1181), P < 0.001 | 0.087 (0.022–0.178), P = 0.009 |
| Poorer SQ → high modified CESD-9 burden → incident digestive disease | Model 3 | 0.0037 (−0.0011–0.0089), P = 0.132 | 0.0738 (0.0461–0.1007), P < 0.001 | 0.0786 (0.0514–0.1046), P < 0.001 | 0.046 (−0.013–0.129), P = 0.132 |
| High modified CESD-9 burden → poorer SQ → incident digestive disease | Model 0 | 0.0227 (0.0147–0.0313), P < 0.001 | 0.0549 (0.0260–0.0853), P < 0.001 | 0.0826 (0.0529–0.1143), P < 0.001 | 0.275 (0.156–0.479), P < 0.001 |
| High modified CESD-9 burden → poorer SQ → incident digestive disease | Model 1 | 0.0210 (0.0133–0.0294), P < 0.001 | 0.0513 (0.0219–0.0816), P < 0.001 | 0.0765 (0.0475–0.1072), P < 0.001 | 0.274 (0.152–0.505), P < 0.001 |
| High modified CESD-9 burden → poorer SQ → incident digestive disease | Model 2 | 0.0210 (0.0133–0.0297), P < 0.001 | 0.0398 (0.0103–0.0701), P = 0.006 | 0.0641 (0.0337–0.0942), P < 0.001 | 0.328 (0.177–0.679), P < 0.001 |
| High modified CESD-9 burden → poorer SQ → incident digestive disease | Model 3 | 0.0165 (0.0100–0.0243), P < 0.001 | 0.0232 (−0.0060–0.0533), P = 0.121 | 0.0410 (0.0116–0.0712), P = 0.006 | 0.402 (0.186–1.436), P = 0.006 |

*Notes*: Analyses included 3,711 participants and 722 incident digestive disease events. ACME and ADE are reported using standard mediation terminology. Modified CESD-9 was calculated by subtracting the restless-sleep item from the CESD-10 total score. High modified CESD-9 burden was defined as modified CESD-9 score ≥9. Poorer SQ was defined as moderate or poor SQ, with good SQ as the reference. Model 0 was unadjusted. Model 1 adjusted for age and sex. Model 2 further adjusted for education level, residence, marital status, BMI category, smoking status, and drinking status. Model 3 included all Model 2 covariates and additionally adjusted for hypertension, diabetes, dyslipidaemia, heart disease, liver disease, lung disease, stroke, kidney disease, asthma, arthritis, pain, memory disorder, and psychiatric disorder. These mediation analyses were exploratory and assumption-dependent and were not interpreted as confirmation of causal pathways. ACME, average causal mediation effect; ADE, average direct effect; PM, proportion mediated; SQ, sleep quality; CESD-9, modified 9-item Center for Epidemiologic Studies Depression Scale; CESD-10, 10-item Center for Epidemiologic Studies Depression Scale; CI, confidence interval; BMI, body mass index.

# Supplementary Table 19. Proportional hazards assumption tests for key Cox models

| **Analysis** | **N** | **Events** | **Exposure** | **Global PH *P* value** | **Exposure-specific PH *P* value** |
| --- | --- | --- | --- | --- | --- |
| SQ category and incident digestive disease | 3,711 | 722 | SQ category | 0.997 | 0.453 |
| CESD-10 depressive symptoms and incident digestive disease | 3,711 | 722 | CESD-10 depressive symptoms | 0.998 | 0.477 |
| Joint SQ and CESD-10 depressive symptoms | 3,711 | 722 | SQ and CESD-10 joint category | 0.959 | 0.158 |
| Continuous SQ score, spline-Cox | 3,711 | 722 | Continuous SQ score | 0.992 | 0.326 |
| Continuous CESD-10 score, spline-Cox | 3,711 | 722 | Continuous CESD-10 score | 0.999 | 0.973 |
| Original joint trajectories of SQ and CESD-10 | 3,711 | 722 | Original SQ–CESD-10 trajectory group | 0.998 | 0.668 |
| High modified CESD-9 burden and incident digestive disease | 3,711 | 722 | High modified CESD-9 burden | 0.998 | 0.888 |
| Joint SQ and high modified CESD-9 burden | 3,711 | 722 | SQ and modified CESD-9 joint category | 0.972 | 0.213 |
| Continuous modified CESD-9 score, spline-Cox | 3,711 | 722 | Continuous modified CESD-9 score | 0.999 | 0.985 |
| Joint trajectories of SQ and modified CESD-9 | 3,711 | 722 | Modified CESD-9 joint trajectory group | 0.998 | 0.692 |

*Notes*: PH, proportional hazards; SQ, sleep quality; CESD-9, modified 9-item Center for Epidemiologic Studies Depression Scale; CESD-10, 10-item Center for Epidemiologic Studies Depression Scale. The proportional hazards assumption was assessed using Schoenfeld residuals in fully adjusted Cox models. The exposure-specific PH *P* value refers to the main exposure term in each model. Modified CESD-9 was calculated by subtracting the restless-sleep item from the CESD-10 total score. No evidence of PH violation was observed for the main exposure terms in these models.

Supplementary Figure 1. Nonlinear association between CESD-10 score and SQ. RCS models showing the dose–response relationship between depressive symptom scores (CESD-10) and SQ score across four models: (A) Crude (unadjusted); (B) Model 1 (adjusted for age and sex); (C) Model 2 (+ socio-demographic variables); (D) Model 3 (+ lifestyle and comorbidities).

*Note*: SQ, sleep quality; CESD-10, 10-item Center for Epidemiologic Studies Depression Scale.

# **Supplementary Figure 2. Sensitivity analyses for the associations of SQ and depressive symptoms with incident digestive disease**.

*Note*: HRs > 1 indicate increased risk of incident digestive disease. HRs, hazard ratios; CIs, confidence intervals; SQ, sleep quality; CESD-10, 10-item Center for Epidemiologic Studies Depression Scale.

Supplementary Figure 3. Association between SQ and modified CESD-9 after excluding the restless-sleep item. Modified CESD-9 was calculated by subtracting the restless-sleep item from the CESD-10 total score. **(A)** Distribution of SQ scores according to high modified CESD-9 burden. **(B)** Density distribution of SQ scores according to high modified CESD-9 burden. **(C)** Distribution of modified CESD-9 scores across SQ categories. **(D)** Density distribution of modified CESD-9 scores across SQ categories. High modified CESD-9 burden was defined as modified CESD-9 score ≥9 for sensitivity analyses only and was not interpreted as a clinical diagnosis of depression. SQ, sleep quality; CESD-9, modified 9-item Center for Epidemiologic Studies Depression Scale; CESD-10, 10-item Center for Epidemiologic Studies Depression Scale.

Supplementary Figure 4. Nonlinear association between modified CESD-9 and SQ. Restricted cubic spline models showing the dose–response relationship between modified CESD-9 score, calculated by subtracting the restless-sleep item from the CESD-10 total score, and SQ score across four models: (A) Crude; (B) Model 1 adjusted for age and sex; (C) Model 2 additionally adjusted for sociodemographic factors; and (D) Model 3 further adjusted for lifestyle factors and comorbidities.

*Note*: SQ, sleep quality; CESD-9, modified 9-item Center for Epidemiologic Studies Depression Scale.

Supplementary Figure 5. Associations of high modified CESD-9 burden with incident digestive disease. Forest plots showing HRs and 95% CIs for incident digestive disease according to high modified CESD-9 burden, defined as CESD-9 ≥9 after excluding the restless-sleep item from the CESD-10 total score. Panels represent four Cox models: **(A)** crude model; **(B)** Model 1 adjusted for age and sex; **(C)** Model 2 further adjusted for sociodemographic and lifestyle factors; and **(D)** Model 3 additionally adjusted for chronic comorbidities. Low modified CESD-9 burden served as the reference group.

*Note*: High modified CESD-9 burden was used only for sensitivity analyses and was not interpreted as a clinical diagnosis of depression. CESD-9, modified 9-item Center for Epidemiologic Studies Depression Scale; HR, hazard ratio; CI, confidence interval.

Supplementary Figure 6. Joint associations of sleep quality and high modified CESD-9 burden with incident digestive disease. Forest plots showing HRs and 95% CIs for incident digestive disease according to joint categories of sleep quality and high modified CESD-9 burden. High modified CESD-9 burden was defined as CESD-9 ≥9 after excluding the restless-sleep item from the CESD-10 total score and was used only for sensitivity analyses, not as a clinical diagnosis of depression. Panels represent four Cox models: **(A)** crude model; **(B)** Model 1 adjusted for age and sex; **(C)** Model 2 further adjusted for sociodemographic and lifestyle factors; and **(D)** Model 3 additionally adjusted for chronic comorbidities. Good SQ with low modified CESD-9 burden served as the reference group. CESD-9, modified 9-item Center for Epidemiologic Studies Depression Scale; HR, hazard ratio; CI, confidence interval; SQ, sleep quality.

Supplementary Figure 7. Dose–response association between modified CESD-9 and incident digestive disease. Restricted cubic spline Cox models showing the association between modified CESD-9 score and incident digestive disease risk. Modified CESD-9 was calculated by subtracting the restless-sleep item from the CESD-10 total score. Panels represent four Cox models: **(A)** crude model; **(B)** Model 1 adjusted for age and sex; **(C)** Model 2 further adjusted for sociodemographic and lifestyle factors; and **(D)** Model 3 additionally adjusted for chronic comorbidities.

*Note*: HRs are shown relative to the reference value defined by the model distribution. CESD-9, modified 9-item Center for Epidemiologic Studies Depression Scale; HR, hazard ratio; CI, confidence interval.

Supplementary Figure 8. Modified CESD-9 joint trajectory sensitivity analysis in relation to incident digestive disease. This sensitivity analysis repeated the original joint trajectory analysis using the same waves and trajectory-construction strategy, while replacing CESD-10 with modified CESD-9 after excluding the restless-sleep item. A single joint trajectory grouping was derived from both sleep quality and modified CESD-9 features. **Group 1** represented participants with stable/low modified CESD-9 and higher SQ; **Group 2** represented participants with a moderate worsening pattern; and **Group 3** represented participants with marked worsening and lower SQ. **(A)** Mean modified CESD-9 trajectories across 2011, 2015, and 2018. **(B)** Forest plot showing HRs and 95% CIs for incident digestive disease across the joint trajectory groups under four Cox models. Group 1 served as the reference group. Model 1 adjusted for age and sex; Model 2 further adjusted for sociodemographic and lifestyle factors; and Model 3 additionally adjusted for chronic comorbidities. CESD-9, modified 9-item Center for Epidemiologic Studies Depression Scale; CI, confidence interval; HR, hazard ratio; SQ, sleep quality.
